# Three mechanisms of language comprehension are revealed through cluster analysis of individuals with language deficits

**DOI:** 10.1038/s41539-024-00284-0

**Published:** 2024-12-03

**Authors:** Andrey Vyshedskiy, Rohan Venkatesh, Edward Khokhlovich, Deniz Satik

**Affiliations:** 1 Boston University, Boston, MA USA; 2 Independent researcher, Newton, MA USA; 3 Harvard University, Cambridge, MA USA

**Keywords:** Language, Intelligence

## Abstract

Analysis of linguistic abilities that are concurrently impaired in individuals with language deficits allows identification of a shared underlying mechanism. If any two linguistic abilities are mediated by the same underlying mechanism, then both abilities will be absent if this mechanism is broken. Clustering techniques automatically arrange these abilities according to their co-occurrence and therefore group together abilities mediated by the same mechanism. This study builds upon the discovery of three distinct mechanisms of language comprehension in 31,845 autistic individuals^1^. The current clustering analysis of a more diverse group of individuals with language impairments resulted in the three mechanisms identical to those found previously: (1) the most-basic command-language-comprehension-mechanism; (2) the intermediate modifier-language-comprehension-mechanism mediating comprehension of color, size, and number modifiers; and (3) the most-advanced syntactic-language-comprehension-mechanism. This discovery calls for mapping of the three empirically-defined language-comprehension-mechanisms in the context of cognitive neuroscience, which is the main goal of this study.

## Introduction

For centuries, numerous scholars have considered the human ability for language as pivotal to the distinctive intelligence of our species. Unraveling the nature and evolution of human language remains a fundamental challenge for the fields of linguistics, cognitive neuroscience, and psychology. The discovery of three distinct mechanisms of language comprehension in our previous study contributes to the ongoing discussion^1^. That discovery emerged from clustering analysis of various language-comprehension abilities in 31,845 autistic individuals. When considering individuals with language deficits one at a time, their linguistic profiles may look different, but when many individuals are studied simultaneously, it becomes increasingly clear that certain abilities appear and disappear in concert (i.e., they co-occur). Clustering techniques automatically arrange abilities according to their similarities (i.e., co-occurrence). If any two linguistic abilities were mediated by the same underlying mechanism, then, when this mechanism is broken, both abilities should be absent and therefore, get clustered into the same group. For instance, consider three types of aphasia. 1) Damage to Wernicke’s area impairs understanding of word meanings and leads to fluid but nonsensical speech^2^. 2) Damage to Broca’s area causes difficulties in word formation (non-fluent speech) and results in the repetition of simple phrases over and over, while comprehension of individual words remains intact^3^. 3) Damage to the arcuate fasciculus white-matter tract, which connects the lateral prefrontal cortex (LPFC, located in the front of the cerebral cortex) with the posterior cortex (the back of the cortex, including the temporal, occipital, and parietal cortices), can disrupt understanding of spatial prepositions, flexible syntax, verb tense, and complex explanations, but does not affect fluent speech or understanding of individual words^4^. Clustering these abilities (understanding of word meanings, speech fluidity, tendency to repeat simple phrases, comprehension of spatial prepositions, flexible syntax, verb tense, and complex explanations) in patients with aphasia can group co-occurring abilities together according to their underlying brain lesion^5,6^. Crucially, the clustering analysis can perform grouping calculations in a data-driven manner without any design or hypothesis, identifying and enumerating underlying mechanisms automatically.

Autistic individuals often display language comprehension deficits^7–13^. Our previous data-driven clustering analysis of fourteen language comprehension abilities in autistic individuals formed three robustly distinct clusters stable across different evaluation methods, across different age groups, and across different time points^1^. The cluster of most-basic abilities, termed “command-language-comprehension,” included knowing the name, responding to “No” or “Stop”, and following some commands. The cluster of intermediate abilities, termed “modifier-language-comprehension,” included understanding color and size modifiers, several modifiers in a sentence, size superlatives, and numbers. The cluster of most-advanced abilities, termed the “syntactic-language-comprehension,” included understanding of spatial prepositions, verb tenses, flexible syntax, possessive pronouns, explanations about people and situations, simple stories, and elaborate fairytales.

Each of the three clusters of co-occurring abilities corresponds to a distinct language mechanism. When the most-advanced syntactic mechanism is disabled, then the complete set of syntactic-language-comprehension-abilities that depend on it is impaired. When both the syntactic and the modifier mechanisms are disabled, then the complete set of syntactic- and modifier-language-comprehension-abilities are impaired. Reassuringly, the additional clustering analysis of participants (rather than abilities) identified three language-comprehension-phenotypes: the most-advanced syntactic-language-comprehension-phenotype included individuals who acquired all three language-comprehension mechanisms – syntactic, modifier, and command; the intermediate modifier-language-comprehension-phenotype included individuals limited to modifier and command mechanisms; and the most-basic command-language-comprehension-phenotype included individuals limited to the command mechanism.

The impetus for the aforementioned study was the theoretical work relating language comprehension to imagination. E.g., to understand an instruction containing spatial prepositions “put the cup under/behind/in front of the table,” most people mentally juxtapose the cup and the table. Similarly, to comprehend the change in meaning when the order of words is changed (termed ‘flexible syntax’), e.g., “the turtle rides/carries the cow” versus “the cow rides/carries the turtle,” most people mentally juxtapose the turtle and the cow. As fairytales commonly describe objects and events that do not exist, a reader has to imagine them by juxtaposing various mental objects from memory. In order to comprehend an unfamiliar syntactic sentence, most people (99.2%) mentally *combine* the subject and the object in front of their mind’s eye^14^. Thus, a connection between language and imagination was hypothesized by many researchers^15–17^.

The mechanism underlying deliberate juxtaposition of two or more mental objects and observing the result of their interaction is called *Prefrontal Synthesis* or PFS^18–20^ (a.k.a. *mental synthesis*^21–23^). Converging evidence suggests that on the neurological level, PFS is mediated by synchronization^24–28^ of neuronal-ensembles that encode those mental objects^29^. In other words, in order to understand a syntactic sentence, such as “the dog lives above the cat and under the monkey,” an interlocutor has to synchronize the neuronal-ensembles encoding all three nouns—the dog, the cat, and the monkey. Neuronal ensembles encoding visual objects are located in the posterior cerebral cortex^30^. The action of their synchronization is mediated by the LPFC^18^ via frontoposterior connections, such as arcuate fasciculus^31^.

Conversely, the mechanism of modifying color or size of a *single* mental object is called *Prefrontal Modification* or PFM (a.k.a., *Prefrontal Analysis*^19^). The neurological difference between PFS and PFM is in the degree of the LPFC ability to control the posterior cortex. In terms of the Dual Visual Stream Theory^32^, the PFM control is limited to the temporal cortex in an area called *ventral pathway*, which encodes the appearance of objects (their shape, form, and color)^19^. PFS adds control of the parietal cortex in an area called *dorsal pathway*, which encodes where objects are in space (their location, motion, depth, and spatial relationship)^33,34^. Consequently, PFM is limited to the control of a *single* mental object’s appearance and PFS can control both mental objects’ appearance and location, enabling juxtaposition of *multiple* mental objects.

Finally, comprehension of commands, such as “take the ball outside” or “bring the cup to daddy,” does not involve any juxtaposition (PFS) or modification (PFM) of mental objects. On the neurological level, comprehension of simple commands relies on recalling existing object-encoding-neuronal-ensembles from memory. E.g., the command “bring the cup to daddy” requires recalling the cup and daddy; there is no modification of a single object-encoding-neuronal-ensemble or synchronization between several object-encoding-neuronal-ensembles.

All three mechanisms – memory recall, PFM, and PFS—are commonly called “imagination,” “symbolic thinking,” “abstract thinking,” “executive function,” or “cognition”^35^. However, there are significant benefits to using the three neurologically defined terms. First, these definitions are much narrower. For example, PFM and PFS are always *voluntary* and *deliberate*, unlike broadly defined “imagination,” which can be *involuntary*, such as during REM-sleep dreaming^19^. The mechanisms of PFM and PFS rely on the LPFC, and patients with the LPFC damage can lose access to these abilities^19,36^. In contrast, *dreaming imagination* does not depend on the LPFC; the LPFC is inactive during sleep^37,38^, and patients with the LPFC damaged do not notice change in their dreams^39^. Moreover, broadly defined “imagination,” “symbolic thinking,” “abstract thinking,” “executive function,” and “cognition” can be mediated by *spontaneous insight*, which primarily depends on the ventromedial prefrontal cortex (vmPFC) rather than the LPFC^40–42^. Neither *involuntary imagination* nor *spontaneous insight* can mediate language comprehension—their mechanisms are too slow and the results are too unpredictable to sustain a typical conversation. When viewed from a neurological perspective, memory recall, PFM, and PFS stand apart from each other and other mechanisms of imagination, and were anticipated to yield three distinct mechanisms of language comprehension^19,36^.

The goals of this study were (1) to validate three language mechanisms using the previously employed 14 language comprehension abilities, and in addition including the previously omitted 15^th^ ability (“Responds to praise”); (2) to focus on participants with normal speech, in order to eliminate potential confusion related to the impact of speech on language comprehension; (3) to explore the relationship between the three empirically-identified language mechanisms and the three tentative mechanisms derived from neurological considerations—memory recall, PFM, and PFS; and (4) to expand the participant pool beyond autistic individuals by including other conditions linked to language impairments: mild language delay, apraxia (a motor speech disorder where individuals struggle to plan and coordinate the movements needed for speech despite normal muscle function), Specific Language Impairment, Sensory Processing Disorder, Social Communication Disorder, Down Syndrome, and Attention-Deficit/Hyperactivity Disorder (ADHD). It was hypothesized that since memory recall, PFM, and PFS are fundamental mechanisms of language comprehension, the inclusion of other pathological conditions with language impairments would result in the same three clusters previously identified in autistic individuals^1^.

## Results

### Clustering analysis of 15 language comprehension abilities

**Fig. 1:**
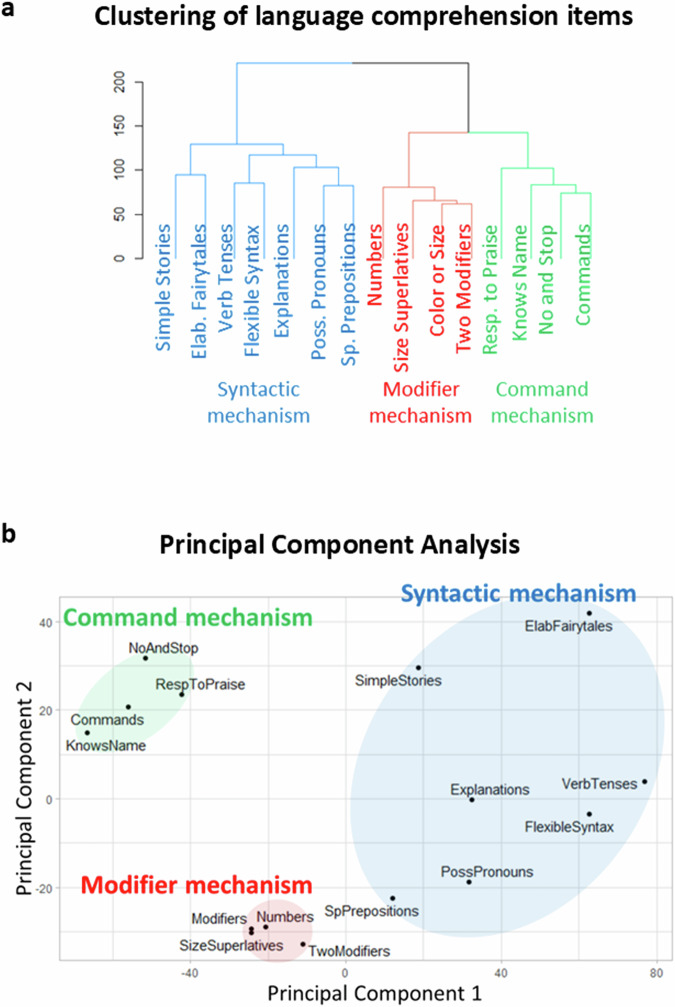
Clustering analysis of language comprehension items. **a** The dendrogram representing the hierarchical clustering of language comprehension abilities. **b** Principal component analysis of the 15 language comprehension abilities shows a clear separation between command, modifier, and syntactic items. Principal component 1 accounts for 35.0% of the variance in the data. Principal component 2 accounts for 11.0% of the variance in the data.

Parents and caregivers evaluated 15 language comprehension abilities (Table 1) in 55,558 individuals with various language deficits (age range of 4 to 22 years). From these participants, we selected those who showed an ability to “use sentences with four or more words” (defined as normal speech; *N* = 17,848, Table 2). To explore co-occurrence of the 15 language comprehension abilities, we used two common clustering methods: Unsupervised Hierarchical Cluster Analysis (UHCA) and Principal Component Analysis (PCA). Figure 1a depicts the dendrogram generated by UHCA. The height of the branches indicates the distance between clusters, which is an indicator of greater dissimilarity. Three clusters have inter-cluster distances that are significantly larger than the distances between subclusters. The right-most cluster includes knowing the name, responding to ‘No’ or ‘Stop’, responding to praise, and following some commands (items 1 to 4 in Table 1). This cluster is identical to the command-language-comprehension-cluster identified by the previous study of 31,845 autistic individuals with the addition of the ‘responds to praise’ item that was not previously analyzed^1^. The cluster in the middle includes understanding color and size modifiers, several modifiers in a sentence, size superlatives, and numbers (items 5 to 8 in Table 1). This cluster is identical to the previously identified modifier-language-comprehension-cluster. The left-most cluster includes understanding of spatial prepositions, verb tenses, flexible syntax, possessive pronouns, explanations about people and situations, simple stories, and elaborate fairy tales (items 9 to 15 in Table 1). This cluster is identical to the previously identified syntactic-language-comprehension-cluster.

**Table 1:** Language comprehension items as they were posed to parents

	Language comprehension items (verbatim)	Abbreviations used in dendrograms
1	Knows own name	Knows Name
2	Responds to ‘No’ or ‘Stop’	No and Stop
3	Responds to praise	Resp. to Praise
4	Can follow some commands	Commands
5	Understands some simple modifiers (i.e., green apple vs. red apple or big apple vs. small apple)	Color or Size / Modifiers
6	Understands several modifiers in a sentence (i.e., small green apple)	Two Modifiers
7	Understands size (can select the largest/smallest object out of a collection of objects)	Size Superlatives
8	Understands NUMBERS (i.e., two apples vs. three apples)	Numbers
9	Understands spatial prepositions (i.e., put the apple ON TOP of the box vs. INSIDE the box vs. BEHIND the box)	Sp. Prepositions
10	Understands verb tenses (i.e., I will eat an apple vs. I ate an apple)	Verb Tenses
11	Understands simple stories that are read aloud	Simple Stories
12	Understands elaborate fairy tales that are read aloud (i.e., stories describing FANTASY creatures)	Elab. Fairy tales
13	Understands possessive pronouns (i.e., your apple vs. her apple)	Poss. Pronouns
14	Understands the change in meaning when the order of words is changed (i.e., understands the difference between ‘a cat ate a mouse’ vs. ‘a mouse ate a cat’)	Flexible Syntax
15	Understands explanations about people, objects or situations beyond the immediate surroundings (e.g., “Mom is walking the dog,” “The snow has turned to water”).	Explanations

Answer choices were as follows: very true (0 points), somewhat true (1 point), and not true (2 points). Items 1 to 4 were assessed as a part of the Autism Treatment Evaluation Checklist (ATEC)^68^; the rest of items were a part of the Mental Synthesis Evaluation Checklist (MSEC)^69^. A lower score indicates better language comprehension ability.

**Table 2:** Participants’ diagnoses as reported by caregivers

	Number of participants	Percent of total	Age, Mean (SD)	Age range	Percent Males
Neurotypical	48	0.3	6.3 (1.3)	4.0–10.1	47.9
Not-diagnosed	4459	25.0	5.6 (1.9)	4.0–21.9	63.5
Mild Language Delay	967	5.4	5.4 (1.4)	4.0–14.4	68.6
Apraxia	76	0.4	6.2 (1.9)	4.1–12.2	67.1
Specific Language Impairment	847	4.7	5.9 (2.0)	4.0–21.3	67.5
Sensory Processing Disorder	149	0.8	6.5 (2.5)	4.0–18.2	64.4
Social Communication Disorder	151	0.8	5.9 (1.7)	4.0–16.1	72.8
Mild Autism Spectrum Disorder (ASD)	7217	40.4	6.3 (2.3)	4.0–21.7	77.1
Moderate ASD	2420	13.6	7.1 (2.8)	4.0–21.9	79.7
Severe ASD	842	4.7	7.5 (3.2)	4.0–20.8	74.6
Down Syndrome	147	0.8	9.2 (4.0)	4.0–20.9	42.9
Other Genetic Disorder	173	1.0	7.7 (3.6)	4.0–19.5	59.5
ADHD	352	2.0	6.5 (2.0)	4.0–14.4	75.3
**Total**	**17848**	**100**	**6.3 (2.4)**	**4.0–21.9**	**72.3**

The PCA (Fig. 1b) also shows clear separation between these three clusters. The command items (knowing the name, responding to ‘No’ or ‘Stop’, responding to praise, and following some commands) are clustered in the top left corner. The modifier items (understanding color and size modifiers, several modifiers in a sentence, size superlatives, and numbers) are clustered in the lower middle. The syntactic items (understanding of spatial prepositions, verb tenses, flexible syntax, possessive pronouns, explanations about people and situations, simple stories, and elaborate fairy tales) are clustered in the top right corner.

The three-cluster solution was stable across multiple seeds as well as consistent across different age groups (4 to 6 years of age, Supplementary Fig. 1; 6 to 12 years of age, Supplementary Fig. 2; 12 to 22 years of age, Supplementary Fig. 3), across different time points (first evaluation, Supplementary Fig. 4; last evaluation, Fig. 1), across different verbal levels (Fig. 1 and Supplementary Fig. 5), across genders (Supplementary Figs. 6 and 7), and across parental education (Supplementary Figs. 8 and 9).

### Language comprehension phenotypes in participants

**Fig. 2:**
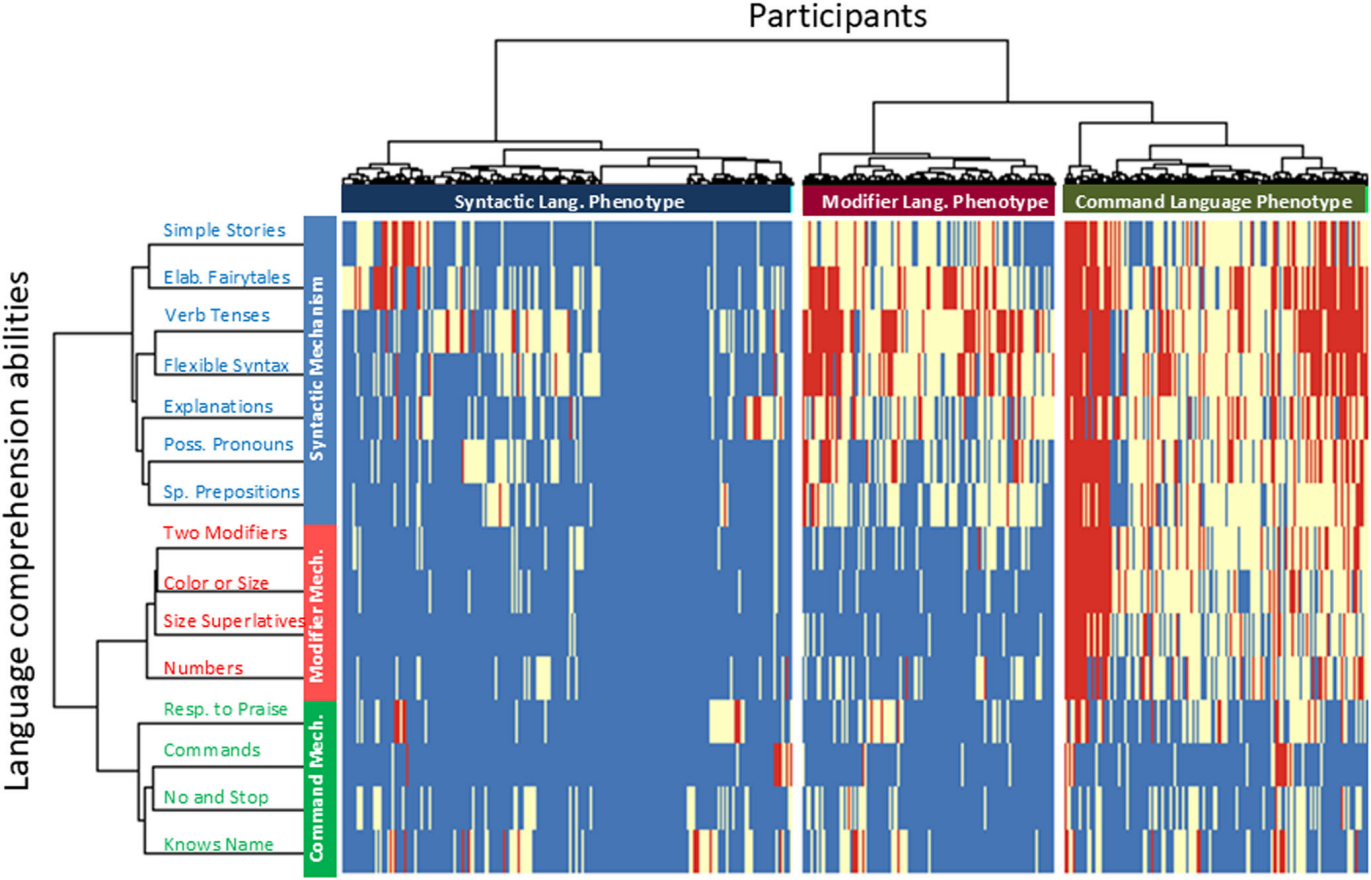
Two-dimensional heatmap relating participants to their language comprehension abilities. The 15 language comprehension abilities are shown as rows. The dendrogram representing language comprehension abilities is shown on the left. Participants are shown as 17,848 columns. The dendrogram representing participants is shown on the top. The green bar labels the command mechanism, the red bar labels the modifier mechanism, and the blue bar labels the syntactic mechanism. The center grid indicates the presence or absence of each ability in each participant: blue signifies the presence of a linguistic ability (“very true” answer), red indicates its absence (“not true” answer), and white represents a partial presence (“somewhat true” answer).

Independently from clustering 15 language comprehension abilities, we utilized UHCA to cluster all 17,848 participants (Fig. 2, the dendrogram on top). This three-cluster solution was stable across different seeds, age groups (4 to 6, 6 to 12, and 6 to 22 years of age, Supplementary Figs. 10–12, respectively, the dendrogram shown on the top), time points (first and last evaluations; Supplementary Fig. 13 and Fig. 2, respectively), across different verbal levels (Fig. 1 and Supplementary Fig. 14), across genders (Supplementary Figs. 15 and 16), and across parental education (Supplementary Figs. 17 and 18).

A two-dimensional heatmap was used to relate participant clusters to language comprehension abilities clusters (Fig. 2). Columns represent the 17,848 participants and rows represent the 15 linguistic abilities. *Blue* indicates the presence of a linguistic ability (parent’s response=*very true*); *white* indicates an intermittent presence of a linguistic ability (parent’s response=*somewhat true*); and *red* indicates the lack of a linguistic ability (parent’s response=*not true*). The three clusters of participants match the three language comprehension mechanisms. The cluster of participants termed “Syntactic Language Phenotype” shows the predominant blue color that indicated good skills across all three (syntactic, modifier, and command) language comprehension mechanisms (44.7% of participants, Table 3). The cluster of participants marked as “Command Language Phenotype” shows the predominant blue color indicating good skills only among the command-mechanism items (30.4%). The third cluster of participants marked “Modifier Language Cluster” shows the predominant blue color indicating good skills only across modifier- and command-mechanisms items (25%). Table 4 shows cluster assignment of participants by their diagnostic category.

**Table 3:** Participant cluster statistics

	Syntactic Language Phenotype	Modifier Language Phenotype	Command Language Phenotype	Total
Number of participants	7975	4455	5418	17848
Percent of Total	44.7	25.0	30.3	100.0
Age, Mean (SD)	6.1 (2.2)	6.2 (2.2)	6.6 (2.8)	6.3 (2.4)
Percent Male	69.5	77.0	72.4	72.3

**Table 4:** Percentage of participants in the three language comprehension phenotypes

	Syntactic Language Phenotype	Modifier Language Phenotype	Command Language Phenotype
Neurotypical	100.0	0.0	0.0
Not-diagnosed	65.6	17.3	17.2
Mild Language Delay	57.4	21.5	21.1
Apraxia	48.7	19.7	31.6
Specific Language Impairment	52.2	21.3	26.6
Sensory Processing Disorder	48.3	24.8	26.8
Social Communication Disorder	43.7	24.5	31.8
Mild ASD	38.8	28.4	32.8
Moderate ASD	24.5	33.9	41.7
Severe ASD	16.2	23.9	60.0
Down Syndrome	37.4	16.3	46.3
Other Genetic Disorder	39.3	19.7	41.0
ADHD	51.4	21.6	27.0

## Discussion

Understanding the mechanisms of human language remains a fundamental challenge of linguistics and cognitive neuroscience. Significant efforts have been invested into uncovering language mechanisms by studying contemporary individuals exhibiting language impairments; e.g., these efforts lead to the discovery of the language-critical FOXP2 gene^43–45^. It is evident that not every contemporary individual acquires every language mechanism. Examining language skills in a population of individuals with language deficits enables researchers to detect different aspects of language manifested in tandem, highlighting patterns where specific abilities emerge and decline in concert and providing insights into the common neurological mechanisms that underpin them. Clustering methods automatically organize language abilities based on their co-occurrence, generating hierarchical structures like dendrograms that illustrate relationships among them. If two linguistic abilities share a common underlying mechanism, their absence due to a disruption of this mechanism results in their clustering together. Importantly, the clustering analysis conducted in this study was unbiased, as both UHCA and PCA were data-driven, devoid of any predetermined design or hypothesis.

This study analyzed 15 language comprehension abilities in 17,848 participants with language impairments but normal speech. First, it confirmed the existence of three robustly distinct language comprehension mechanisms discovered in autistic individuals^1^. Compared to the previous investigation, this study expanded the participant pool to include all individuals whose parents have submitted assessments, regardless of children’s diagnosis. Furthermore, in order to eliminate potential confusion related to the impact of speech, this study only enrolled participants with normal speech. The three identified mechanisms were as follows: (1) the command mechanism mediated comprehension of one’s name, responding to ‘No’ or ‘Stop,’ responding to praise, and following simple commands (Fig. 1); (2) the modifier mechanism mediated comprehension of simple color/size modifiers, understanding of several modifiers in a sentence, understanding of size superlatives, and number comprehension; and (3) the syntactic mechanism mediated comprehension of spatial prepositions, verb tenses, flexible syntax, possessive pronouns, explanations, simple stories, and elaborate fairy tales.

This study also confirmed previous discovery of three *language comprehension phenotypes* (Fig. 2). Participants in the syntactic-language-phenotype-cluster acquired all three language mechanisms: syntactic, modifier, and command. All neurotypical participants (controls) were clustered into this group. Participants in the modifier-language-phenotype-cluster acquired only modifier and command mechanisms and did not acquire the syntactic mechanism. Participants in the command-language-phenotype-cluster acquired only the command mechanism and did not acquire the syntactic and the modifier mechanisms.

As expected, all neurotypical children were clustered into the syntactic cluster (Table 4). Among the not-diagnosed participants, who include a high proportion of typically developing individuals, the majority exhibited the syntactic language phenotype (65.6%), with the lowest proportion found in the command language phenotype (17.2%). Participants with mild language delay followed a similar pattern, with 57.4% in the syntactic phenotype and 21.1% in the command phenotype. A higher prevalence of the syntactic language phenotype was also observed in participants diagnosed with specific language impairment (52.2%), ADHD (51.4%), apraxia (48.7%), and sensory processing disorder (48.3%).

Participants diagnosed with more severe conditions exhibit a lower proportion of the syntactic phenotype. Those with severe autism had the smallest proportion clustered in the syntactic phenotype (16.2%) and the highest proportion in the command phenotype (60.0%). They are followed by participants with moderate autism, of whom only 24.5% fell into the syntactic phenotype, while 41.7% were clustered in the command phenotype. They were followed by participants diagnosed with Down syndrome, of whom only 37.4% fell into the syntactic phenotype, while 46.3% were clustered in the command phenotype.

The prevalence of the command phenotype increased with Autism Spectrum Disorder (ASD) severity: the command phenotype was observed in 32.8% of individuals diagnosed with mild ASD, in 41.7% of individuals diagnosed with moderate ASD, and in 60.0% of individuals diagnosed with severe ASD (Table 4). Conversely, the prevalence of the syntactic phenotype decreased with ASD severity: the syntactic phenotype was observed in 38.8% of individuals diagnosed with mild ASD, in 24.5% of individuals diagnosed with moderate ASD, and in 16.2% of individuals diagnosed with severe ASD.

Future studies should explore the genetic correlates of the three language phenotypes. However, it is evident that multiple different genetic abnormalities can lead to an inferior phenotype. The functionality of each linguistic mechanism depends on the optimized state of the entire central nervous system, including fine-tuned axonal connections, synaptic configuration, myelination, etc. This optimized state can be conceptualized as *an attractor state*, defined as a state toward which a child tends to progress from a range of starting conditions of the nervous system^46,47^. Any single neurological abnormality (synaptic impairment, reduced myelination, cell-surface proteins dysfunction, etc.) can render the most-advanced attractor neurological state inaccessible for a developing child. If the nervous system cannot optimize for the most-advanced syntactic-language-comprehension-phenotype, it may optimize for the modifier-language-comprehension-phenotype; and if it cannot optimize for the modifier-language-comprehension-phenotype, it may still optimize for the command-language-comprehension-phenotype. Our results suggest that a variety of genetic conditions can delay or prevent the achievement of the most-advanced syntactic-language-comprehension-phenotype attractor state.

**Fig. 3:**
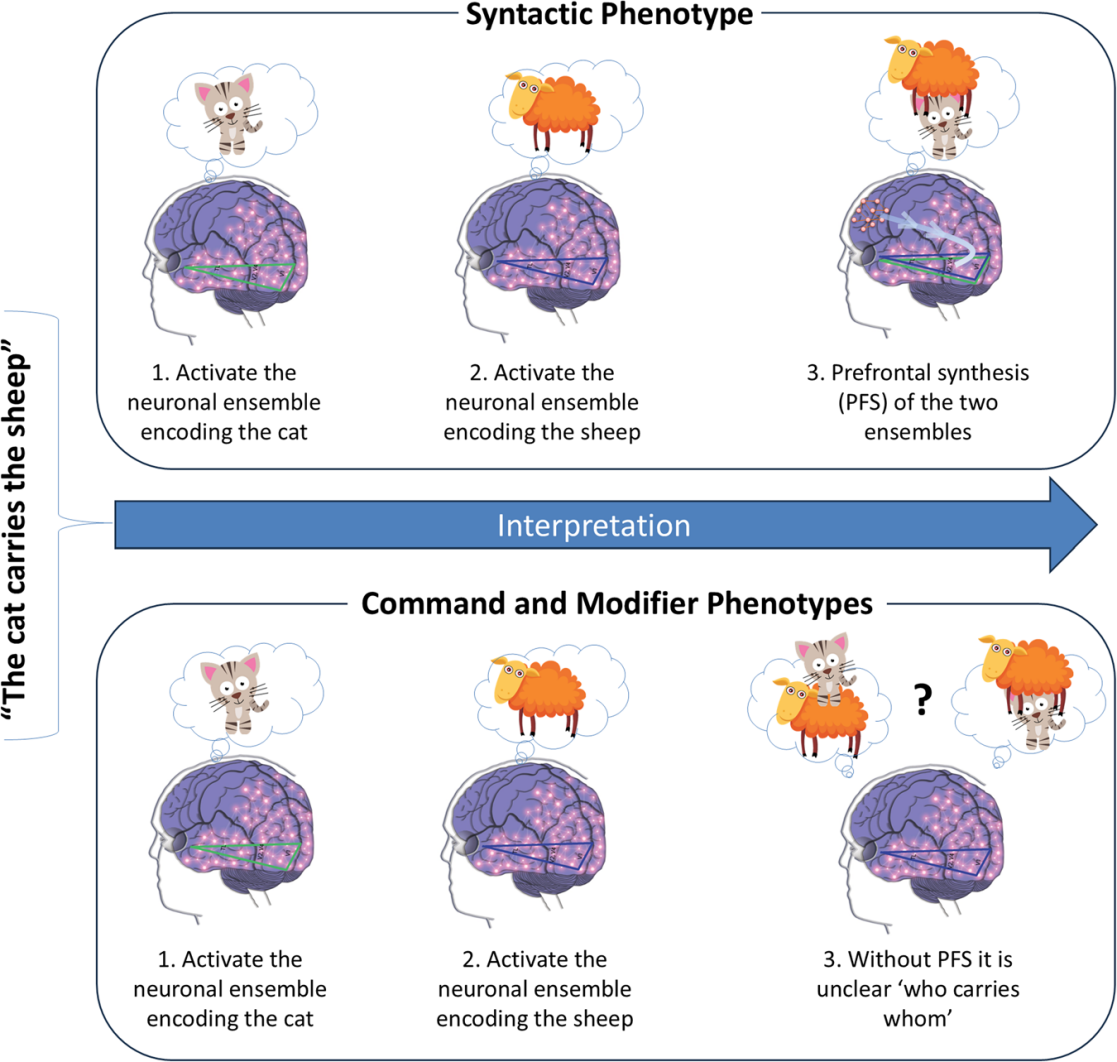
Differences in comprehension between language phenotypes. Individuals with the syntactic phenotype interpret the sentence “the cat carries the sheep” by recalling the cat, the sheep, and juxtaposing the two objects using the prefrontal synthesis (PFS) mechanism. In contrast, individuals with the command and modifier phenotypes do not acquire the PFS mechanism, preventing them from understanding the relational aspect of sentences—specifically, ‘who carries whom’.

One of the most interesting aspects of the study involves interpretation of its results in terms of cognitive neuroscience. Even simple syntactic sentences, such as “the cat carries the sheep,” require a complex neurological machinery for their interpretation. First, the Wernicke’s area matches the word ‘cat’ resulting in activation of the neuronal ensemble encoding the cat (i.e., recall of the cat’s image from memory). Second, the Wernicke’s area matches the word ‘sheep’ resulting in activation of the neuronal ensemble encoding the sheep (i.e., recall of the sheep’s image from memory). This memory recall is followed by the LPFC juxtaposing the two recalled objects mediated by the process of prefrontal synthesis (PFS)^18,48^ governed by the verb ‘carries’ (Fig. 3). If the verb ‘rides’ was used instead, the LPFC would re-arrange the scene, constructing the mental image with the cat on top of the sheep. Thus, the interpretation of this sentence includes at least two neurological mechanisms: (1) recall of the two neuronal ensembles by the Wernicke’s area and (2) their juxtaposition by the LPFC (the PFS mechanism discussed in the introduction).

**Fig. 4:**
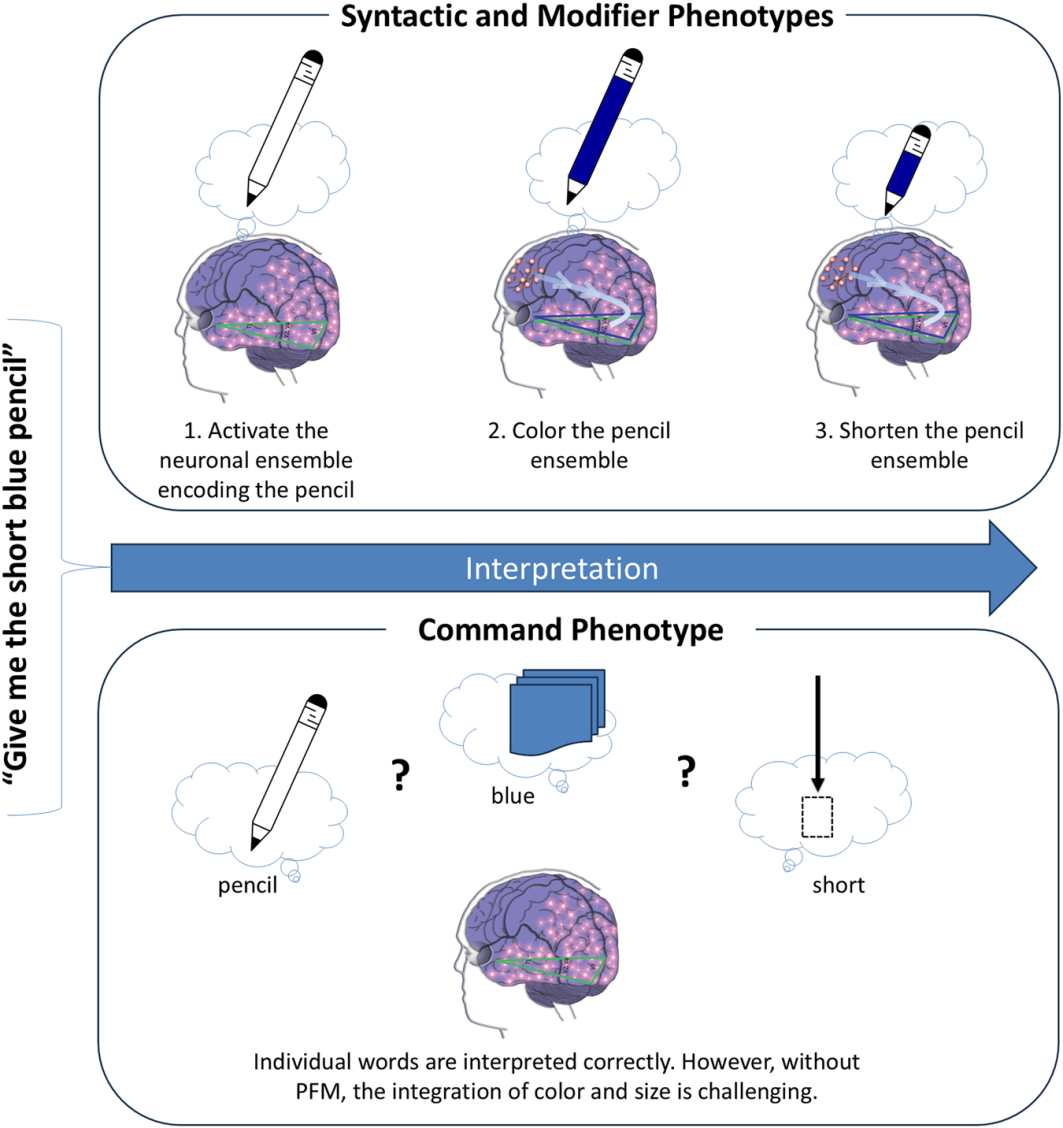
Differences in comprehension between language phenotypes. Individuals with the syntactic and modifier phenotypes can follow the instruction “give me the short blue pencil” by recalling a pencil, and modifying its attributes, such as color and length, using the prefrontal modification (PFM) mechanism. In contrast, individuals with the command phenotype can understand individual words, but do not acquire the PFM mechanism. As a result, they struggle to identify the correct pencil among short/long straws, pencils, and Legos of different colors. This difficulty in integrating multiple elements into a coherent whole is known as *stimulus overselectivity*, *tunnel vision*, or *the lack of multi-cue responsivity*^77–80^.

Conversely, understanding of a sentence “give me the short blue pencil” does not involve PFS, since it describes a single object (pencil). The Wernicke’s area first matches the word ‘pencil’ resulting in activation of the neuronal ensemble encoding the pencil (i.e., recall of the pencil’s image from memory); then the LPFC modifies the pencil-encoding neuronal ensemble to imbue it with the smaller size and the blue color using the process of prefrontal modification (Fig. 4; the PFM mechanism discussed in the introduction).

Finally, understanding of the command sentence type is mediated by neither PFS nor PFM. To interpret the sentence “take the cup to daddy,” an individual with the command-language-comprehension-phenotype only needs to recall the words ‘cup’ and ‘daddy.’ The action is implicit—no other reasonable action is possible between the cup and daddy. Neurologically, this instruction is understood by the Wernicke’s area activation of the neuronal ensemble encoding the cup (i.e., recall of the cup’s image from memory); then by the Wernicke’s activation of the neuronal ensemble encoding daddy (i.e., recall of the daddy’s image from memory). The two separate neuronal ensembles remain in working memory. This interpretation involves no object combination or object modification (i.e., neither PFS nor PFM).

Accordingly, from the neurological perspective, all sentence types that require a combination of two or more mental objects are expected to be mediated by the PFS mechanism and therefore cluster together; all sentence types that require modification of a single object’s color or size are expected to be mediated by the PFM mechanism and cluster into the second group; and all sentence types that do not require modification or juxtaposition of objects are expected to be mediated by memory recall and cluster into the third group. These predictions were borne out of data.

Out of fifteen sentence types investigated in this study, seven types involved a combination of two or more mental objects and therefore, were expected to be mediated by the PFS mechanism. Sentences with spatial prepositions (Table 1, item 9) require juxtaposition of a subject and an object in a manner governed by spatial prepositions (on, under, behind, etc.) in order to understand them. Semantically-reversible sentences that change their meaning when the order of words is changed (i.e., ‘a cat ate a mouse’ vs. ‘a mouse ate a cat’; Table 1, item 14) also require juxtaposition a subject and an object in order to understand them. Simple stories and elaborate fairytales (Table 1, items 11 and 12) usually describe objects and events that do not exist, and a reader has to imagine them by juxtaposing various mental objects from memory. Similarly, understanding explanations about people, objects, or situations beyond the immediate surroundings (e.g., “Mom is walking the dog,” “The snow has turned to water,” Table 1, item 15) commonly require juxtaposition of a subject and an object. Understanding of sentences with possessive pronouns (Table 1, items 13) also requires a combination of two mental objects: e.g., “her apple” requires a combination of a female and an apple; “mom’s sock” requires a combination of mom and a sock. Finally, understanding verb tenses (i.e., I will eat an apple vs. I ate an apple, Table 1, items 10) also requires a combination of a subject and an object governed by the verb. All these sentence types require a combination of two or more mental objects (Table 1, items 9 to 15) mediated by the PFS mechanism and thus expected to cluster together. This prediction was borne out of data; all seven sentence types have been clustered into the most-advanced syntactic mechanism (Fig. 1). None of the sentence types that do not require combination of two or more mental objects (Table 1, items 1 to 8) have been clustered into the most-advanced syntactic mechanism. This suggests that the most-advanced syntactic mechanism is mediated by the PFS.

Sentence types that require modification of attributes of a single mental object include the following: understanding some simple modifiers (i.e., green apple vs. red apple or big apple vs. small apple, Table 1, item 5); understanding several modifiers in a sentence (i.e., small green apple, Table 1, item 6); and understanding size (can select the largest/smallest object out of a collection of objects, Table 1, item 7). Accordingly, these sentence types were predicted to be mediated by the PFM mechanism and thus expected to cluster together. This prediction was borne out of data (Fig. 1, the modifier mechanism). Additionally, understanding small numbers (Table 1, item 8; e.g., two apples vs. three apples,) clustered within the modifier mechanism, suggesting that small number interpretation is mediated by the PFM mechanism as well.

Sentences that do not require modification and juxtaposition of mental objects and therefore do not rely on the PFM and PFS mechanisms include the following: knowing own name (Table 1, item 1); responding to ‘No’ or ‘Stop’ (Table 1, item 2); responding to praise (Table 1, item 3); and following some commands (Table 1, item 4). These sentence types were predicted to cluster separately from those relying on syntactic and modifier mechanisms, the phenomenon borne out of the data. This finding suggests that the command-language-comprehension-mechanism requires neither the PFS nor the PFM mechanisms.

It is important to note that in individuals with the syntactic-language-comprehension-phenotype, PFS can accompany interpretation of command sentences: neurotypical adults can visualize a novel scenario of “taking the cup to daddy.” However, this does not imply that interpreting command sentences always requires PFS. Individuals with the command-language-comprehension-phenotype can accurately interpret commands, such as “take the ball outside” and “bring the cup to daddy” simply by recalling one object at a time, without PFM or PFS.

An additional mechanism of interpretation of syntactic sentences that does not rely on PFS is routinization. Once a sentence type has been routinized, an interlocutor only needs to recall the objects mentioned in the sentence and follow the algorithm. For instance, given the instruction “Put the red cup inside the green cup,” a routinized algorithm would involve (1) lifting the cup mentioned first and (2) inserting it into the cup mentioned second. This method achieves the correct result by relying solely on memory recall of the red cup and green cups; without requiring PFS.

Accordingly, routinized interpretation of syntactic sentences can be taught to individuals who do not acquire PFS. However, the routinized method is rigid and supports only canonical-word-order processing (i.e., “put the red cup inside the green cup”). It fails with non-canonical-word-order instructions (i.e., “inside the green cup, put the red cup”). This limitation is evident from our recent study that compared typical children (~5 years of age) with autistic adolescents (~18 years of age)^49^. Typical children understood both canonical and non-canonical instructions equally well. In contrast, autistic adolescents were able to complete the canonical stacking cups task but only half of them were able to complete the same task under the condition of non-canonical-word-order (participants were not time-limited and the instruction was repeated multiple times). Those who failed usually selected the correct cups, but assembled them incorrectly (Supplementary Movie 1). Autistic adolescents who failed to understand non-canonical sentences also failed to understand spatial prepositions and complex explanations, suggesting that they did not acquire PFS^49^. Their success with canonical instructions likely reflects routinization through extensive Applied Behavior Analysis (ABA) therapy, which often includes tasks like stacking cups.

Several limitations of the study should be acknowledged. Epidemiological studies leveraging app users as participants offer access to a substantial number of individuals, but they do have some evident drawbacks, such as reliance on parental reports and parent-provided diagnoses. On one hand, parents may be prone to wishful thinking and their reports may overestimate their children’s abilities^50^. On the other hand, parents possess a deep understanding of their children, which can be particularly valuable for assessing syntactic language skills that can be challenging to evaluate in a clinical setting. Furthermore, several previous studies have indicated that parent reports of language abilities align closely with direct assessments conducted by clinicians^51–53^. Our own database studies also support the consistency and reliability of parent reports^54–56^.

The diagnosis selection dialog was designed to be as easy as possible for caregivers. First, caregivers were permitted to select only one diagnosis. Second, the presence or absence of autism was emphasized. For example, the option for ADHD was labeled: “No autism. Diagnosed with ADHD or ADD.” Consequently, while this design simplified the selection process, it also means we cannot rule out the possibility that language impairments may be associated with participants diagnosed with ADHD.

Another limitation of the study is that the majority of participants were in the 4 to 8 years of age range. That is the nature of data collection procedure through language therapy app. Future studies should aim to include a broader age range to capture data from older participants as well.

The parent survey used in this study assessed the understanding of fifteen sentence types. It is theoretically possible that if more sentence types were used in the survey, these types could cluster into some additional mechanisms. However, based on the cognitive neuroscience interpretation, we predict that interpretation of all sentence types falls into the three mechanisms: syntactic, modifier, and command. For instance, although recursive sentence structures were not included in the survey, we anticipate they would fall under the syntactic mechanism. Chomsky explains recursion in spatial reasoning with examples such as “((((the hole) in the tree) in the glade) by the stream)” and suggests that there is no limit to such embeddings of place concepts within place concepts^57^. This process of recursion, as used in spatial reasoning, aligns with the syntactic mechanism. Neither the command-, nor modifier-language-comprehension-mechanisms enable recursive sentences, as colors, sizes, and numbers alone cannot facilitate construction of infinite recursive sentences. The syntactic-language-comprehension phenotype, however, does encompass the comprehension of recursion; e.g., comprehension of spatial prepositions, which is a part of the syntactic phenotype is sufficient for understanding Chomsky’s example of recursion in spatial reasoning.

This study focused on verbal participants, which reduced the sample size from 55,558 to 17,848 and skewed the selection towards individuals with higher linguistic abilities. Consequently, some clusters have shown an increased heterogeneity. For example, the syntactic cluster in Fig. 1b (based on the smaller sample of verbal participants) appears less homogeneous compared to the syntactic cluster in Supplementary Fig. 5 (derived from the full sample). Despite the smaller sample size, this approach offers significant advantages, particularly in minimizing potential confounding effects of speech difficulties on language comprehension.

The discovery of three mechanisms of language comprehension can help advance research in related fields. Our results underscore the limitations of broad terms, such as “symbolic thinking,” “abstract thinking,” “executive function,” “cognition,” “visual integration,” and “imagination” in adequately describing the mechanisms underlying syntactic language comprehension. Without clearly defining the subcomponents of symbolic thinking – PFS, PFM, and memory recall – we would not have been able to interpret the three empirically identified language-comprehension mechanisms (Fig. 1).

In linguistics, the idea that language comprehension consists of several components has been articulated by the Merge hypothesis^58,59^. Merge is defined as a cognitive operation that takes two linguistic units (e.g., ‘blue’ and ‘cat’) and forms a set (‘blue cat’), which can then be further combined with additional linguistic units. The field of linguistics has coalesced on the hypothesis that unbounded Merge is a uniquely human ability, and this operation is the generative engine underlying the ability of humans to communicate infinite expressions^60,61^. A key goal of the Merge hypothesis is to link linguistics with neurology by identifying a uniquely human neurological mechanism underlying Merge. Our clustering analysis of language abilities in individuals with language impairments provides a path to explore this connection. Unbounded Merge is able to mediate the comprehension of syntactic (Table 1, items 9–15) and modifier (Table 1, items 5–8) structures, but not command structures, which are limited by definition to comprehension of single words (Table 1, items 1–4). This distinction positions Merge as a separator between modifier and syntactic abilities on one hand, and command abilities on the other. Our finding of a large cluster of individuals limited to command abilities supports the Merge hypothesis by confirming that command abilities are mediated by a mechanism separate from Merge. Moreover, our results refine the hypothesis by distinguishing two subcomponents of Merge. 25% of participants in our study acquired the modifier mechanism without developing the syntactic mechanism. This suggests a division within the Merge operation: “simple Merge,” which in our study underlies the comprehension of color, size, and number modifiers and is mediated by PFM, and “complex Merge,” which underlies the comprehension of syntactic structures and is mediated by PFS.

Future studies should explore the genetic correlates of the three language comprehension phenotypes. Identifying genes that hinder the acquisition of syntactic language could pave the way for new treatment discoveries. Moreover, pinpointing language-critical genes may enhance our understanding of human language evolution^57,62,63^.

In the absence of pharmaceutical treatments capable of improving a language phenotype, language therapy remains the primary approach. Language therapists can benefit from a more precise characterization of language comprehension phenotypes. First, the current terminology primarily describes an individual’s communication level in terms of their use of spoken language, categorizing individuals as *nonverbal*, *minimally verbal*, or *verbal*. However, this study highlights the importance of assessing communication levels based on their language comprehension phenotype in addition to the spoken language level (all study participants were verbal and still fell into the three distinct language comprehension phenotypes). Second, with a clearer understanding of phenotypes, language interventions can be more effectively tailored to each individual. For instance, those in the command phenotype may benefit more from exercises focused on color, size, and number modifiers, as syntactic exercises might be too challenging for them. Third, improved language comprehension assessments can be developed (at the level reached by typical children aged 2 to 4 years), facilitating earlier recognition of comprehension difficulties and a better tracking of children’s progress. This, in turn, could lead to earlier interventions and more focused therapy, which over time may enable children to achieve higher language comprehension phenotypes.

## Methods

### Study participants

Participants were children and adolescents using a language therapy app that was made freely available at all major app stores in September 2015^48,64–67^. The app provides various structured language comprehension therapy exercises and is primarily used by parents of children with language impairments. Once the app was downloaded, caregivers were asked to register and to provide demographic details, including the child’s diagnosis and age. Caregivers consented to pseudonymized data analysis and completed a 133-item questionnaire (77-item Autism Treatment Evaluation Checklist^68^, Supplementary Tables 1 to 4; 20-item Mental Synthesis Evaluation Checklist (MSEC)^69^, Supplementary Table 5; 10-item screen time checklist^55^; 25-item diet checklist^70^; and 1-item parent education survey) approximately every three months.

All fifteen available *language comprehension* items from the 133-item questionnaire were included in the cluster analysis (Table 1). Compared to the previous study^1^, item 3 “Responds to praise” was added to clustering analysis. Given that most praise is delivered verbally, this item assesses an individual’s language comprehension, justifying its inclusion in the cluster analysis. Answer choices were as follows: very true (0 points), somewhat true (1 point), and not true (2 points). A lower score indicates better language comprehension ability.

The inclusion criteria for this study remained consistent with those of the previous study^1^: absence of seizures (which commonly result in intermittent, unstable language comprehension deficits^71^), absence of serious and moderate sleep problems (which are also associated with intermittent, unstable language comprehension deficits^56^), age range of 4 to 22 years (the lower age cutoff was chosen to ensure that participants were exposed to complete set of sentence structures listed in Table 1^72^; the upper age cutoff was chosen to avoid analysis of participants who may be linguistically declining due to aging). The previous study was limited to individuals diagnosed with Autism Spectrum Disorder (ASD). This study included all participants who submitted their assessments through the app (*N* = 55,510). Table 2 reports participants’ diagnoses as communicated by caregivers. Autism level (mild/Level 1, moderate/Level 2, or severe/Level 3) was reported by caregivers. Pervasive Developmental Disorder and Asperger Syndrome were combined with mild autism for analysis as recommended by DSM-5^73^. A good reliability of such parent-reported diagnosis has been previously demonstrated^54^. As a control we have added 48 neurotypical children of 4 to 10 years of age, whose data were previously collected for a different study^72^ by approaching parents on a community online site and asking if they would be willing to complete a Google form. Participants identified by caregivers as ‘no autism, normally developed child’ are labeled in Table 2 as the ‘Not-diagnosed’ group in order to differentiate them from the 48 neurotypical controls.

The goal of this study was to focus on participants with normal speech, in order to eliminate potential confusion related to the impact of speech. Therefore, from these 55,558 individuals, we selected those who showed an ability to “use sentences with four or more words” (parents had to respond ‘very true’ to this question; the complete list of options was ‘very true,’ ‘somewhat true,’ and ‘not true’). Since normal speech is usually associated with mild ASD, this group is overrepresented of the three levels of ASD severity (Table 2).

When caregivers have completed several evaluations, the last evaluation was used for analysis. Thus, the study included a total of 17,848 participants, the average age was 6.3 ± 2.4 years (range of 4 to 21.9 years), 72.3% participants were males. The education level of participants’ parents was the following: 91.7% with at least a high school diploma, 71.4% with at least college education, 39.0% with at least a master’s, and 6.8% with a doctorate. All caregivers consented to pseudonymized data analysis and publication of results. The study was conducted in compliance with the Declaration of Helsinki^74^. Using the Department of Health and Human Services regulations found at 45 CFR 46.101(b)(4), the Biomedical Research Alliance of New York (BRANY) LLC Institutional Review Board (IRB) determined that this research project is exempt from IRB oversight (IRB File # 22-12-205-1120). The data were accessed on 5th of April, 2023. Supplementary Movie 1 is reprinted with permission from ref. ^75^. Authors have obtained written parental consent to publish the video.

### Statistics and reproducibility

UHCA was performed using Ward’s agglomeration method with a Euclidean distance metric. Clustering analysis was data-driven without any design or hypothesis. Two-dimensional heatmap was generated using the “pheatmap” package of R, freely available language for statistical computing^76^. Code is available from the corresponding author upon reasonable request.

As a control we calculated UHCA and PCA of the 15 language comprehension abilities along with the “hyperactivity” item graded on the scale of four: ‘not a problem’, ‘mild problem,’ ‘moderate problem,’ and ‘severe problem.’ From a neurological perspective, hyperactivity is not related to any particular language ability and therefore should cluster into its own group. As expected, both UHCA and PCA clustered the hyperactivity item into its own group at a significant distance from the three language clusters, validating the effectiveness of both clustering techniques (Supplementary Fig. 19).

## Supplementary information

SUPPLEMENTAL MATERIAL

Movie 1

## Data Availability

De-identified raw data from this manuscript are available from the corresponding author upon reasonable request.

## Code availability

Code is available from the corresponding author upon reasonable request.

## References

[1] Vyshedskiy, A., Venkatesh, R. & Khokhlovich, E. Are there distinct levels of language comprehension in autistic individuals – cluster analysis. *Npj Ment. Health Res*. **3**, 19 (2024). 10.1038/s41539-024-00284-0 PMC11612420 39622810

[2] Ardila, A., Bernal, B. & Rosselli, M. The role of Wernicke’s area in language comprehension. *Psychol. Neurosci.* **9**, 340 (2016).

[3] Andrews, J. P. et al. Dissociation of Broca’s area from Broca’s aphasia in patients undergoing neurosurgical resections. *J. Neurosurg.* **138**, 847–857 (2022). 35932264 10.3171/2022.6.JNS2297 PMC9899289

[4] Dragoy, O., Akinina, Y. & Dronkers, N. Toward a functional neuroanatomy of semantic aphasia: a history and ten new cases. *Cortex* **97**, 164–182 (2017). 28277283 10.1016/j.cortex.2016.09.012

[5] Fromm, D., Greenhouse, J., Pudil, M., Shi, Y. & MacWhinney, B. Enhancing the classification of aphasia: a statistical analysis using connected speech. *Aphasiology* **36**, 1492–1519 (2022). 36457942 10.1080/02687038.2021.1975636 PMC9708051

[6] Kertesz, A. & Phipps, J. B. Numerical taxonomy of aphasia. *Brain Lang.* **4**, 1–10 (1977). 832101 10.1016/0093-934x(77)90001-3

[7] Barsotti, J. et al. Grammatical comprehension in italian children with autism spectrum disorder. *Brain Sci.* **10**, 510 (2020). 32748841 10.3390/brainsci10080510 PMC7464622

[8] Boucher, J. Research review: structural language in autistic spectrum disorder–characteristics and causes. *J. Child Psychol. Psychiatry* **53**, 219–233 (2012). 22188468 10.1111/j.1469-7610.2011.02508.x

[9] Mitchell, S. et al. Early language and communication development of infants later diagnosed with autism spectrum disorder. *J. Dev. Behav. Pediatr.* **27**, S69–S78 (2006). 16685188 10.1097/00004703-200604002-00004

[10] Hudry, K. et al. Preschoolers with autism show greater impairment in receptive compared with expressive language abilities. *Int. J. Lang. Commun. Disord.* **45**, 681–690 (2010). 20102259 10.3109/13682820903461493

[11] Seol, K. I. et al. A comparison of receptive-expressive language profiles between toddlers with autism spectrum disorder and developmental language delay. *Yonsei Med. J.* **55**, 1721–1728 (2014). 25323912 10.3349/ymj.2014.55.6.1721 PMC4205715

[12] Ellis Weismer, S., Lord, C. & Esler, A. Early language patterns of toddlers on the autism spectrum compared to toddlers with developmental delay. *J. Autism Dev. Disord.* **40**, 1259–1273 (2010). 20195735 10.1007/s10803-010-0983-1 PMC2941531

[13] Eigsti, I. M., Bennetto, L. & Dadlani, M. B. Beyond pragmatics: morphosyntactic development in autism. *J. Autism Dev. Disord.* **37**, 1007–1023 (2007). 17089196 10.1007/s10803-006-0239-2

[14] Dance, C. J., Ipser, A. & Simner, J. The prevalence of aphantasia (imagery weakness) in the general population. *Conscious. Cognit.* **97**, 103243 (2022). 34872033 10.1016/j.concog.2021.103243

[15] Papeo, L., Corradi-Dell’Acqua, C. & Rumiati, R. I. “She” is not like “I”: the tie between language and action is in our imagination. *J. Cognit. Neurosci.* **23**, 3939–3948 (2011). 21671735 10.1162/jocn_a_00075

[16] Dor, D. From experience to imagination: language and its evolution as a social communication technology. *J. Neurolinguist.* **43**, 107–119 (2017).

[17] Reuland, E. Language and imagination: evolutionary explorations. *Neurosci. Biobehav. Rev.* **81**, 255–278 (2017). 28041788 10.1016/j.neubiorev.2016.12.017

[18] Vyshedskiy, A. & Dunn, R. Mental synthesis involves the synchronization of independent neuronal ensembles. *Res. Ideas Outcomes* **1**, e7642 (2015).

[19] Vyshedskiy, A. Neuroscience of imagination and implications for hominin evolution. *J. Curr. Neurobiol.* **10**, 89–109 (2019).

[20] Vyshedskiy, A. Voluntary and involuntary imagination: neurological mechanisms, developmental path, clinical implications, and evolutionary trajectory. *Evol. Stud. Imagin. Cult.* **4**, 1–17 (2020).

[21] Rowton, S. J. *Inaugural Dissertation on the Inseparable Co-Operation of Sense and Intellect for Arriving at Cognitions*, (M’Gowan & Danks, 1864).

[22] Finke, R. A. & Slayton, K. Explorations of creative visual synthesis in mental imagery. *Mem. Cognit.* **16**, 252–257 (1988). 3393086 10.3758/bf03197758

[23] Pearson, D. G., Logie, R. H. & Gilhooly, K. J. Verbal representations and spatial manipulation during mental synthesis. *Eur. J. Cognit. Psychol.* **11**, 295–314 (1999).

[24] Hirabayashi, T. & Miyashita, Y. Dynamically modulated spike correlation in monkey inferior temporal cortex depending on the feature configuration within a whole object. *J. Neurosci.* **25**, 10299–10307 (2005). 16267238 10.1523/JNEUROSCI.3036-05.2005 PMC6725794

[25] Herrmann, C. S., Lenz, D., Junge, S., Busch, N. A. & Maess, B. Memory-matches evoke human gamma-responses. *BMC Neurosci.* **5**, 1–8 (2004). 15084225 10.1186/1471-2202-5-13 PMC419345

[26] Sehatpour, P. et al. A human intracranial study of long-range oscillatory coherence across a frontal–occipital–hippocampal brain network during visual object processing. *Proc. Natl Acad. Sci. USA* **105**, 4399–4404 (2008). 18334648 10.1073/pnas.0708418105 PMC2393806

[27] Singer, W. & Gray, C. M. Visual feature integration and the temporal correlation hypothesis. *Annu. Rev. Neurosci.* **18**, 555–586 (1995). 7605074 10.1146/annurev.ne.18.030195.003011

[28] Hipp, J. F., Engel, A. K. & Siegel, M. Oscillatory synchronization in large-scale cortical networks predicts perception. *Neuron* **69**, 387–396 (2011). 21262474 10.1016/j.neuron.2010.12.027

[29] Carrillo-Reid, L. & Yuste, R. Playing the piano with the cortex: role of neuronal ensembles and pattern completion in perception and behavior. *Curr. Opin. Neurobiol.* **64**, 89–95 (2020). 32320944 10.1016/j.conb.2020.03.014 PMC8006069

[30] Koch, C., Massimini, M., Boly, M. & Tononi, G. Neural correlates of consciousness: progress and problems. *Nat. Rev. Neurosci.* **17**, 307 (2016). 27094080 10.1038/nrn.2016.22

[31] Wilson, S. M. et al. Syntactic processing depends on dorsal language tracts. *Neuron* **72**, 397–403 (2011). 22017996 10.1016/j.neuron.2011.09.014 PMC3201770

[32] Ray, D., Hajare, N., Roy, D. & Banerjee, A. Large-scale functional integration, rather than functional dissociation along dorsal and ventral streams, underlies visual perception and action. *J. Cognit. Neurosci.* **32**, 847–861 (2020). 31933430 10.1162/jocn_a_01527

[33] Lee, K. H. et al. Neural correlates of superior intelligence: stronger recruitment of posterior parietal cortex. *Neuroimage* **29**, 578–586 (2006). 16122946 10.1016/j.neuroimage.2005.07.036

[34] Goodale, M. A. & Milner, A. D. Separate visual pathways for perception and action. *Trends Neurosci.* **15**, 20–25 (1992). 1374953 10.1016/0166-2236(92)90344-8

[35] Tattersall, I. *Becoming Human: Evolution and Human Uniqueness*, (Houghton Mifflin Harcourt, 1999).

[36] Vyshedskiy, A., Dunn, R. & Piryatinsky, I. Neurobiological mechanisms for nonverbal IQ tests: implications for instruction of nonverbal children with autism. *Res. Ideas Outcomes* **3**, e13239 (2017).

[37] Braun, A. R. et al. Regional cerebral blood flow throughout the sleep-wake cycle. *Brain* **120**, 1173–1197 (1997). 9236630 10.1093/brain/120.7.1173

[38] Siclari, F. et al. The neural correlates of dreaming. *Nat. Neurosci.* **20**, 872 (2017). 28394322 10.1038/nn.4545 PMC5462120

[39] Solms, M. *The Neuropsychology of Dreams: A Clinico-Anatomical Study*, (L. Erlbaum, 1997). 10.1016/s1364-6613(98)01166-8 21227155

[40] Salvi, C., Bricolo, E., Kounios, J., Bowden, E. & Beeman, M. Insight solutions are correct more often than analytic solutions. *Think. Reason.* **22**, 443–460 (2016). 27667960 10.1080/13546783.2016.1141798 PMC5035115

[41] Wagner, U., Gais, S., Haider, H. & Born, J. Sleep inspires insight. *Nature* **427**, 352–355 (2004). 14737168 10.1038/nature02223

[42] Bartoli, E. et al. Default mode network electrophysiological dynamics and causal role in creative thinking. *Brain* **147**, 3409–3425 (2024). 38889248 10.1093/brain/awae199 PMC11449134

[43] Chen, P. et al. The language development via FOXP2 in autism spectrum disorder: a review. *Curr. Pharm. Des.* **26**, 4789–4795 (2020). 32912122 10.2174/1381612826666200909141108

[44] Enard, W. et al. Molecular evolution of FOXP2, a gene involved in speech and language. *Nature* **418**, 869–872 (2002). 12192408 10.1038/nature01025

[45] Toma, C. et al. Analysis of two language-related genes in autism: a case–control association study of: FOXP2: And: CNTNAP2. *Psychiatr. Genet.* **23**, 82–85 (2013). 23277129 10.1097/YPG.0b013e32835d6fc6

[46] Khona, M. & Fiete, I. R. Attractor and integrator networks in the brain. *Nat. Rev. Neurosci.* **23**, 744–766 (2022). 36329249 10.1038/s41583-022-00642-0

[47] Dor, D. & Jablonka, E. Plasticity and canalization in the evolution of linguistic communication: an evolutionary developmental approach. *Evol. Hum. Lang. Biolinguistic Perspect.* **135**, 147 (2010).

[48] Vyshedskiy, A. et al. Novel prefrontal synthesis intervention improves language in children with autism. *Healthcare* **8**, 566 (2020). 33339269 10.3390/healthcare8040566 PMC7765988

[49] Vyshedskiy, A. et al. Novel Linguistic Evaluation of Prefrontal Synthesis (LEPS) test measures prefrontal synthesis acquisition in neurotypical children and predicts high-functioning versus low-functioning class assignment in individuals with autism. *Appl. Neuropsychol. Child*10.1080/21622965.2020.1758700 (2020). 10.1080/21622965.2020.1758700 32420749

[50] Scattone, D., Raggio, D. J. & May, W. Comparison of the vineland adaptive behavior scales, and the bayley scales of infant and toddler development. *Psychol. Rep.* **109**, 626–634 (2011). 22238860 10.2466/03.10.PR0.109.5.626-634

[51] Miller, L. E., Perkins, K. A., Dai, Y. G. & Fein, D. A. Comparison of parent report and direct assessment of child skills in toddlers. *Res. Autism Spectr. Disord.* **41**, 57–65 (2017). 28919924 10.1016/j.rasd.2017.08.002 PMC5599144

[52] Dale, P. S., Bates, E., Reznick, J. S. & Morisset, C. The validity of a parent report instrument of child language at twenty months. *J. Child Lang.* **16**, 239–249 (1989). 2760125 10.1017/s0305000900010394

[53] Netson, R. et al. A Comparison of Parent Reports, the Mental Synthesis Evaluation Checklist (MSEC) and the Autism Treatment Evaluation Checklist (ATEC), with the Childhood Autism Rating Scale (CARS). *Pediatr. Rep.* **16**, 174–189 (2024). 38535512 10.3390/pediatric16010016 PMC10975750

[54] Jagadeesan, P., Kabbani, A. & Vyshedskiy, A. Parent-reported assessment scores reflect ASD severity level in 2- to 7- year-old children. *Children* **9**, 701 (2022). 35626878 10.3390/children9050701 PMC9139745

[55] Fridberg, E., Khokhlovich, E. & Vyshedskiy, A. Watching Videos and Television Is Related to a Lower Development of Complex Language Comprehension in Young Children with Autism. *Healthcare* **9**, 423 (2021). 10.3390/healthcare9040423 PMC8067341 33917303

[56] Levin, J., Khokhlovich, E. & Vyshedskiy, A. Longitudinal developmental trajectories in young autistic children presenting with sleep problems, compared to those presenting without sleep problems, gathered via parent-report using a mobile application. *Res. Autism Spectr. Disord.* **97**, 102024 (2022).

[57] Fitch, W. T., Hauser, M. D. & Chomsky, N. The evolution of the language faculty: clarifications and implications. *Cognition* **97**, 179–210 (2005). 16112662 10.1016/j.cognition.2005.02.005

[58] Chomsky, N. *The minimalist program*, (MIT Press, 1995).

[59] Chomsky, N. Minimalist inquiries: The framework. *Step by Step: Essays on Minimalist Syntax in Honor of Howard Lasnik.* 89–155 (2000).

[60] Martins, P. T. & Boeckx, C. Language evolution and complexity considerations: the no half-Merge fallacy. *PLoS Biol.* **17**, e3000389 (2019). 31774810 10.1371/journal.pbio.3000389 PMC6880980

[61] Tanaka, K. et al. Merge-generability as the key concept of human language: evidence from neuroscience. *Front. Psychol.* **10**, 2673 (2019). 31849777 10.3389/fpsyg.2019.02673 PMC6895067

[62] Vyshedskiy. Language evolution to revolution: the leap from rich-vocabulary non-recursive communication system to recursive language 70,000 years ago was associated with acquisition of a novel component of imagination, called Prefrontal Synthesis, enabled by a mutation that slowed down the prefrontal cortex maturation simultaneously in two or more children – the Romulus and Remus hypothesis. *Res. Ideas Outcomes* **5**, e38546 (2019).

[63] Dediu, D. & Levinson, S. C. Neanderthal language revisited: not only us. *Curr. Opin. Behav. Sci.* **21**, 49–55 (2018).

[64] Vyshedskiy, A. & Dunn, R. Mental Imagery Therapy for Autism (MITA)-An Early Intervention Computerized Brain Training Program for Children with ASD. *Autism Open Access* **5**, 2 (2015).

[65] Dunn, R. et al. Comparison of performance on verbal and nonverbal multiple-cue responding tasks in children with ASD. *Autism Open Access* **7**, 218 (2017).

[66] Dunn, R. et al. Tablet-based cognitive exercises as an early parent-administered intervention tool for toddlers with autism - evidence from a field study. *Clin. Psychiatry* **3**, 1–8 (2017).

[67] Dunn, R. et al. Children with autism appear to benefit from parent-administered computerized cognitive and language exercises independent of the child’s age or autism severity. *Autism Open Access* **7**, 10.4172/2165-7890.1000217 (2017).

[68] Rimland, B. & Edelson, S. M. Autism treatment evaluation checklist (ATEC). *Autism Res. Inst. San Diego CA* (1999).

[69] Braverman, J., Dunn, R. & Vyshedskiy, A. Development of the Mental Synthesis Evaluation Checklist (MSEC): A Parent-Report Tool for Mental Synthesis Ability Assessment in Children with Language Delay. *Children* **5**, 62 (2018). 29783788 10.3390/children5050062 PMC5977044

[70] Acosta, A., Khokhlovich, E., Reis, H. & Vyshedskiy, A. Dietary factors impact developmental trajectories in young autistic children. *J. Autism Dev. Disord*. 10.1007/s10803-023-06074-8 (2023). 10.1007/s10803-023-06074-8 37584768

[71] Forman, P., Khokhlovich, E. & Vyshedskiy, A. Longitudinal developmental trajectories in young autistic children presenting with seizures, compared to those presenting without seizures, gathered via parent-report using a mobile application. *J. Dev. Phys. Disabil*. 10.1007/s10882-022-09851-y (2022).

[72] Arnold, M. & Vyshedskiy, A. Combinatorial language parent-report score differs significantly between typically developing children and those with Autism Spectrum Disorders. *J. Autism Dev. Disord*. 10.1007/s10803-022-05769-8 (2022). 10.1007/s10803-022-05769-8 36315319

[73] American Psychiatric Association. *Diagnostic and Statistical Manual of Mental Disorders (DSM-5®)*. (American Psychiatric Pub, 2013).

[74] World Medical Association. World Medical Association Declaration of Helsinki: ethical principles for medical research involving human subjects. *JAMA* **310**, 2191–2194 (2013). 24141714 10.1001/jama.2013.281053

[75] Vyshedskiy, A. Imagination in Autism: a chance to improve early language therapy. *Healthcare* **9**, 63 (2021). 33440627 10.3390/healthcare9010063 PMC7826637

[76] R Foundation for Statistical Computing. R: A language and environment for statistical computing. (2021).

[77] Schreibman, L. Diagnostic features of autism. *J. Child Neurol.* **3**, S57–S64 (1988). 3058787 10.1177/0883073888003001s11

[78] Lovaas, O. I., Koegel, R. L. & Schreibman, L. Stimulus overselectivity in autism: a review of research. *Psychol. Bull.* **86**, 1236–1254 (1979). 515280

[79] Ploog, B. O. Stimulus overselectivity four decades later: a review of the literature and its implications for current research in autism spectrum disorder. *J. Autism Dev. Disord.* **40**, 1332–1349 (2010). 20238154 10.1007/s10803-010-0990-2

[80] Dube, W. V. et al. Stimulus overselectivity in autism, Down syndrome, and typical development. *Am. J. Intellect. Dev. Disabil.* **121**, 219–235 (2016). 27119213 10.1352/1944-7558-121.3.219 PMC4850837

