# Supplementary material for: Three mechanisms of language comprehension are revealed through cluster analysis of individuals with language deficits
Source: NPJ Sci Learn. 2024 Dec 3;9:74. doi: 10.1038/s41539-024-00284-0 (PMC11612420; doi:10.1038/s41539-024-00284-0)
Supplement: Supplementary file 1 — SUPPLEMENTAL MATERIAL [file 41539_2024_284_MOESM1_ESM.pdf]

Supplementary Material

Supplementary Table 1: Autism Treatment Evaluation Checklist (ATEC) [1], subscale 1: Speech/Language/Communication. The answers choices were: not true, somewhat true, very true.

1. Knows own name
2. Responds to ‘No’ or ‘Stop’
3. Can follow some commands
4. Can use one word at a time (No!, Eat, Water, etc.)
5. Can use 2 words at a time (Don't want, Go home)
6. Can use 3 words at a time (Want more milk)
7. Knows 10 or more words
8. Can use sentences with 4 or more words
9. Explains what he/she wants
10. Asks meaningful questions
11. Speech tends to be meaningful/relevant
12. Often uses several successive sentences
13. Carries on fairly good conversation
14. Has normal ability to communicate for his/her age

Supplementary Table 2: ATEC subscale 2: Sociability**.** The answers choices were: not true, somewhat true, very true.

1. Seems to be in a shell – you cannot reach him/her
2. Ignores other people
3. Pays little or no attention when addressed
4. Uncooperative and resistant
5. No eye contact
6. Prefers to be left alone
7. Shows no affection
8. Fails to greet parents
9. Avoids contact with others
10. Does not imitate
11. Dislikes being held/cuddled
12. Does not share or show
13. Does not wave ‘bye bye’
14. Disagreeable/not compliant
15. Temper tantrums
16. Lacks friends/companions
17. Rarely smiles
18. Insensitive to other's feelings
19. Indifferent to being liked
20. Indifferent if parent(s) leave

Supplementary Table 3: ATEC subscale 3: Sensory/Cognitive awareness**.** The answers choices were: not true, somewhat true, very true.

1. Responds to own name
2. Responds to praise
3. Looks at people and animals
4. Looks at pictures (and T.V.)
5. Does drawing, coloring, art
6. Plays with toys appropriately
7. Appropriate facial expression
8. Understands stories on T.V.
9. Understands explanations
10. Aware of environment
11. Aware of danger
12. Shows imagination
13. Initiates activities
14. Dresses self
15. Curious, interested
16. Venturesome - explores
17. “Tuned in” — Not spacey
18. Looks where others are looking

Supplementary Table 4: ATEC subscale 4: Health/Physical/Behavior**.** The answers choices were: not a problem, minor problem, moderate problem, and serious problem.

1. Bed-wetting
2. Wets pants/diapers
3. Soils pants/diapers
4. Diarrhea
5. Constipation
6. Sleep problems
7. Eats too much/too little
8. Extremely limited diet
9. Hyperactive
10. Lethargic
11. Hits or injures self
12. Hits or injures others
13. Destructive
14. Sound-sensitive
15. Anxious/fearful
16. Unhappy/crying
17. Seizures
18. Obsessive speech
19. Rigid routines
20. Shouts or screams
21. Demands sameness
22. Often agitated
23. Not sensitive to pain
24. “Hooked” or fixated on certain objects/topics
25. Repetitive movements (stimming, rocking, etc.)

Supplementary Table 5: Mental Synthesis Evaluation Checklist (MSEC) [2]. The answers choices were: not true, somewhat true, very true.

1. Understands simple stories that are read aloud
2. Understands elaborate fairy tales that are read aloud (i.e. stories describing FANTASY creatures)
3. Draws a VARIETY of RECOGNIZABLE images (objects, people, animals, etc.)
4. Can draw a NOVEL image following YOUR description (e.g. a three-headed horse)
5. Engages in a VARIETY of make-believe activities (such as: playing house, playing with toy soldiers, building forts and castles, etc.)
6. Understands some simple modifiers (i.e. green apple vs. red apple or big apple vs. small apple)
7. Understands several modifiers in a sentence (i.e. small green apple)
8. Understands size (can select the largest/smallest object out of a collection of objects)
9. Understands possessive pronouns (i.e. your apple vs. her apple)
10. Understands spatial prepositions (i.e. put the apple ON TOP of the box vs. INSIDE the box vs. BEHIND the box)
11. Understands verb tenses (i.e. I will eat an apple vs. I ate an apple)
12. Understands the change in meaning when the order of words is changed (i.e. understands the difference between 'a cat ate a mouse' vs. 'a mouse ate a cat')
13. Understands NUMBERS (i.e. two apples vs. three apples)
14. Can perform simple arithmetic: 2 + 3 = ?
15. Can add larger numbers: 7 + 6 = ?
16. Can perform simple subtraction: 3 – 2 = ?
17. Can subtract larger numbers: 15 – 7 = ?
18. Can perform simple multiplication: 2 × 2 = ?
19. Can multiply larger numbers: 6 × 7 = ?
20. Understands explanations about people, objects or situations beyond the immediate surroundings (e.g., “Mom is walking the dog,” “The snow has turned to water”)

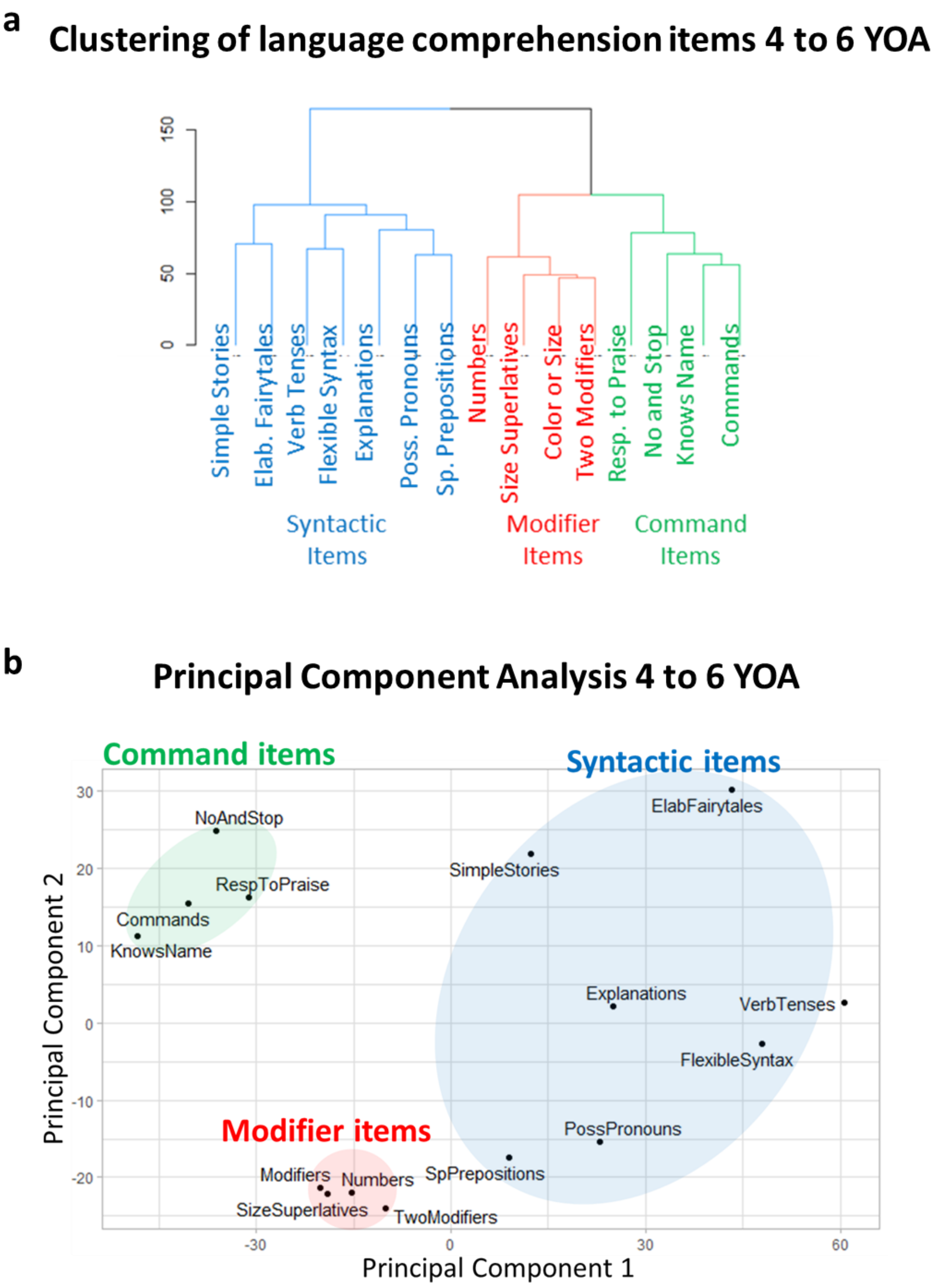

Supplementary Figure 1. Clustering analysis of language comprehension items limited to 10,454 participants (4 to 6 years of age. (a) The dendrogram representing the hierarchical clustering of language comprehension abilities. (b) Principal component analysis of the 15 language comprehension abilities shows a clear separation between command, modifier, and syntactic items. Principal component 1 accounts for 34.1% of the variance in the data. Principal component 2 accounts for 10.7% of the variance in the data.

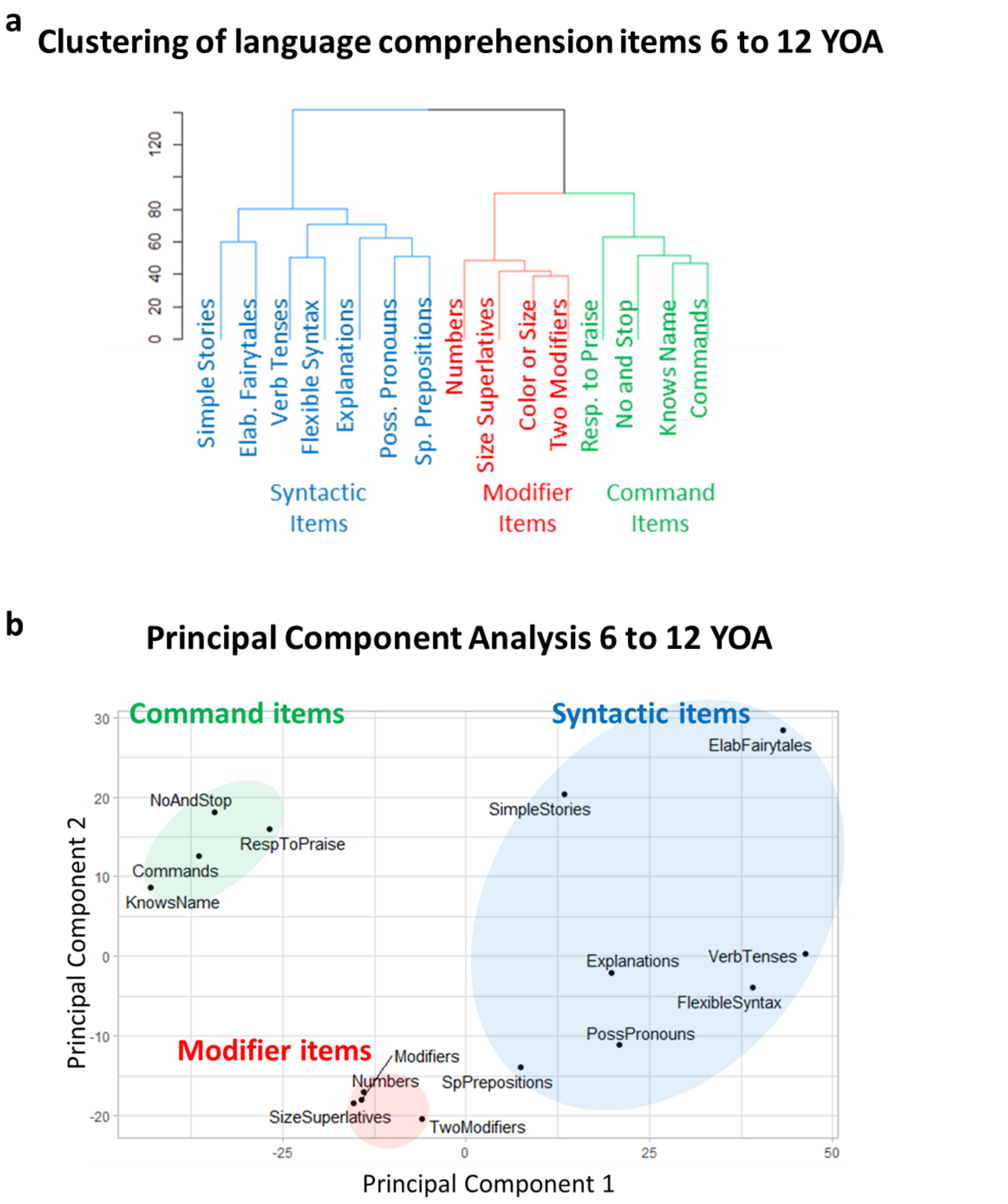

Supplementary Figure 2. Clustering analysis of language comprehension items limited to 6,745 participants (6 to 12 years of age). (a) The dendrogram representing the hierarchical clustering of language comprehension abilities. (b) Principal component analysis of the 15 language comprehension abilities shows a clear separation between command, modifier, and syntactic items. Principal component 1 accounts for 36.9% of the variance in the data. Principal component 2 accounts for 11.2% of the variance in the data.

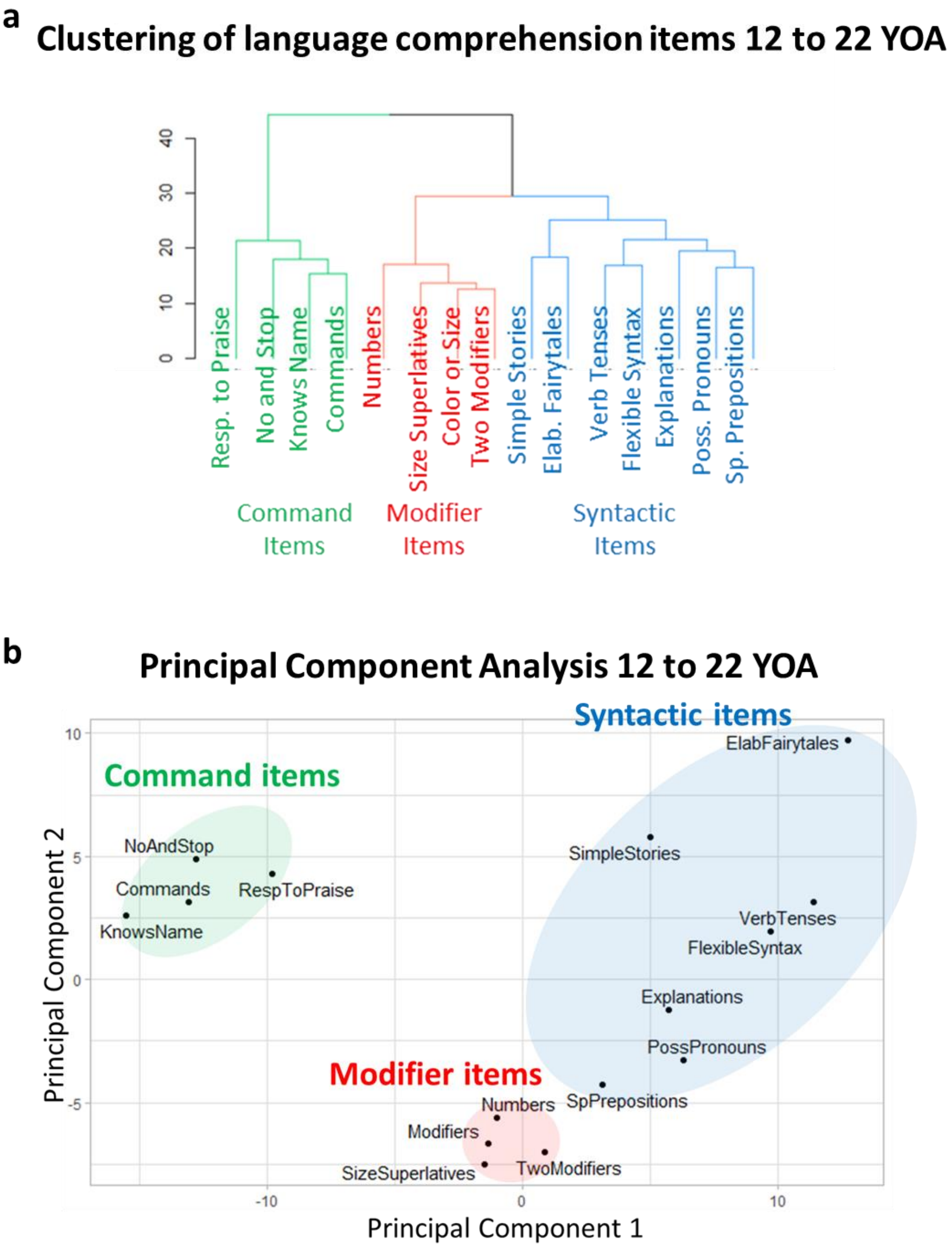

Supplementary Figure 3. Clustering analysis of language comprehension items limited to 633 participants (12 to 22 years of age). (a) The dendrogram representing the hierarchical clustering of language comprehension abilities. (b) Principal component analysis of the 15 language comprehension abilities shows a clear separation between command, modifier, and syntactic items. Principal component 1 accounts for 34.2% of the variance in the data. Principal component 2 accounts for 12% of the variance in the data.

a

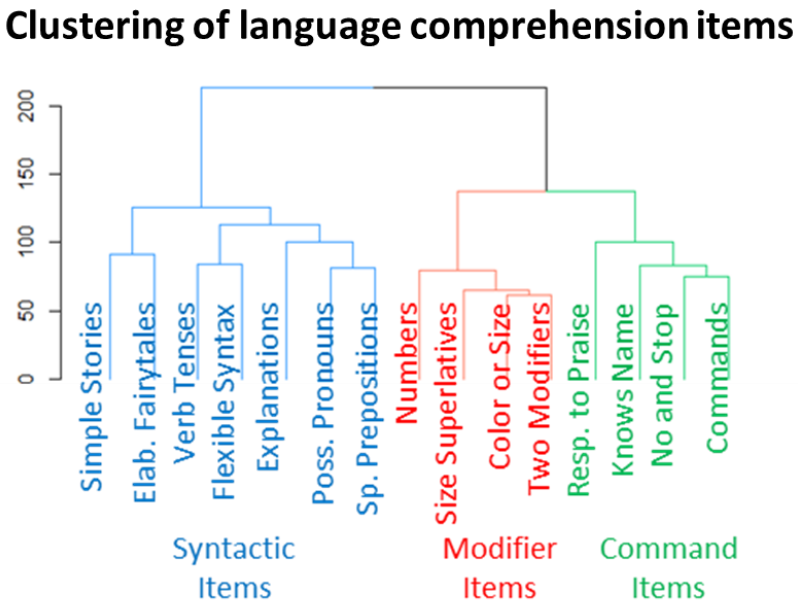

b

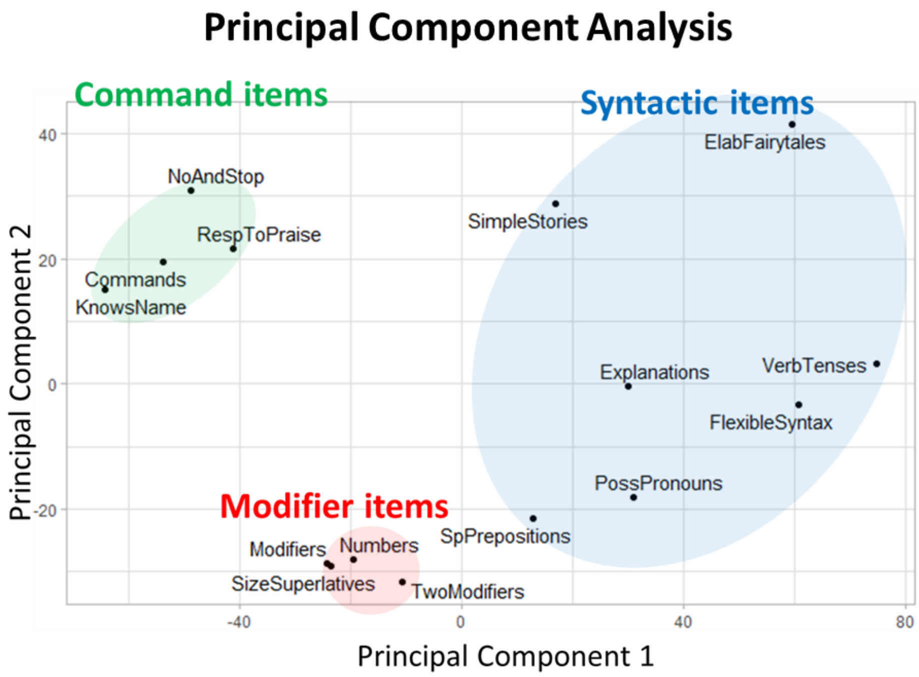

Supplementary Figure 4. Clustering analysis of language comprehension items (17,848 participants, 4 to 22 years of age, **first evaluation**). (a) The dendrogram representing the hierarchical clustering of language comprehension abilities. (b) Principal component analysis of the 15 language comprehension abilities shows a clear separation between command, modifier, and syntactic items. Principal component 1 accounts for 34.3% of the variance in the data. Principal component 2 accounts for 10.9% of the variance in the data.

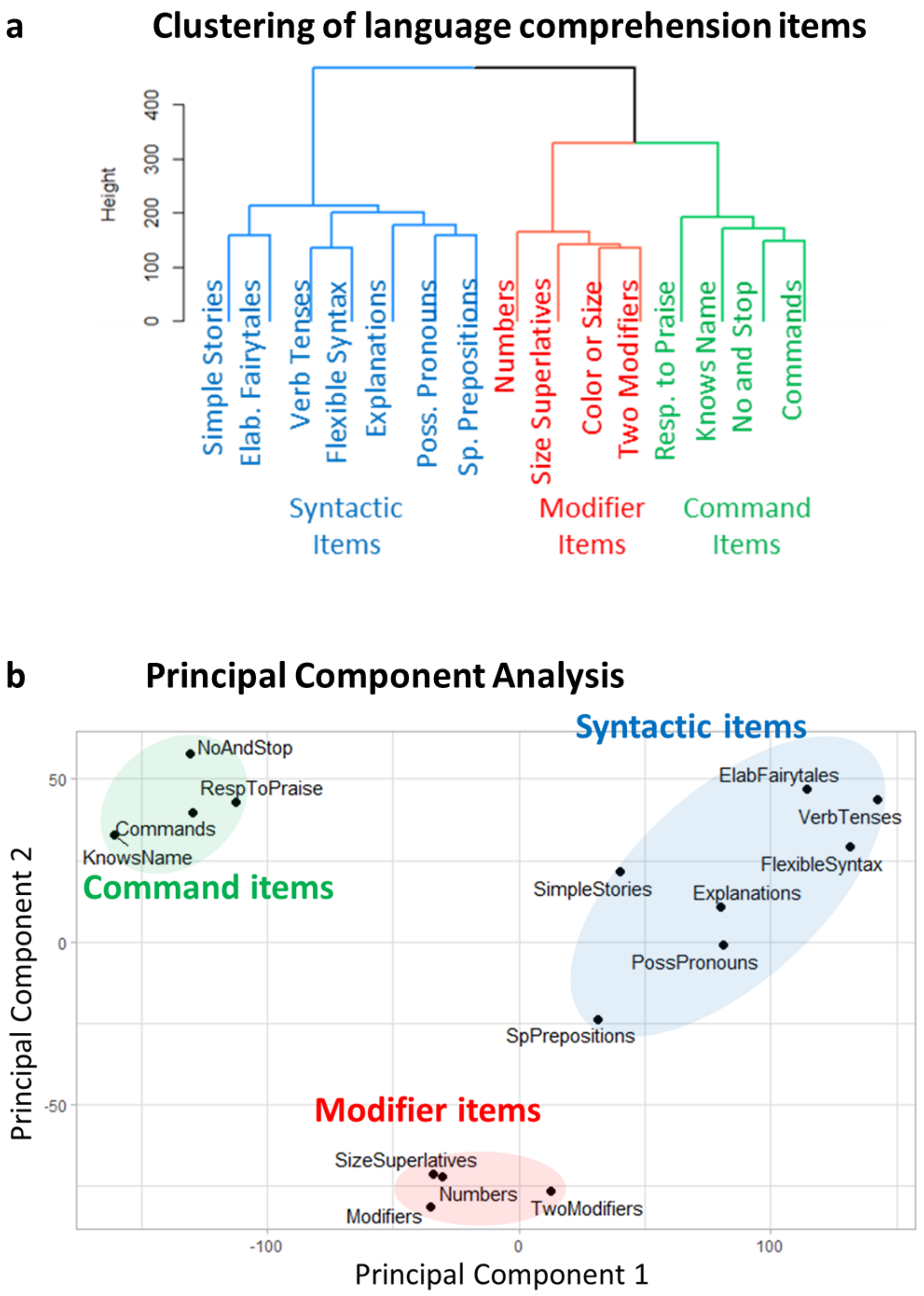

Supplementary Figure 5. Clustering analysis of language comprehension items (**all participants were included in the analysis independent of their verbal level N=55,558**, 4 to 22 years of age). (a) The dendrogram representing the hierarchical clustering of language comprehension abilities. (b) Principal component analysis of the 15 language comprehension abilities shows a clear separation between command, modifier, and syntactic items. Principal component 1 accounts for 45.4% of the variance in the data. Principal component 2 accounts for 10.9% of the variance in the data.

a **Clustering of language comprehension items**

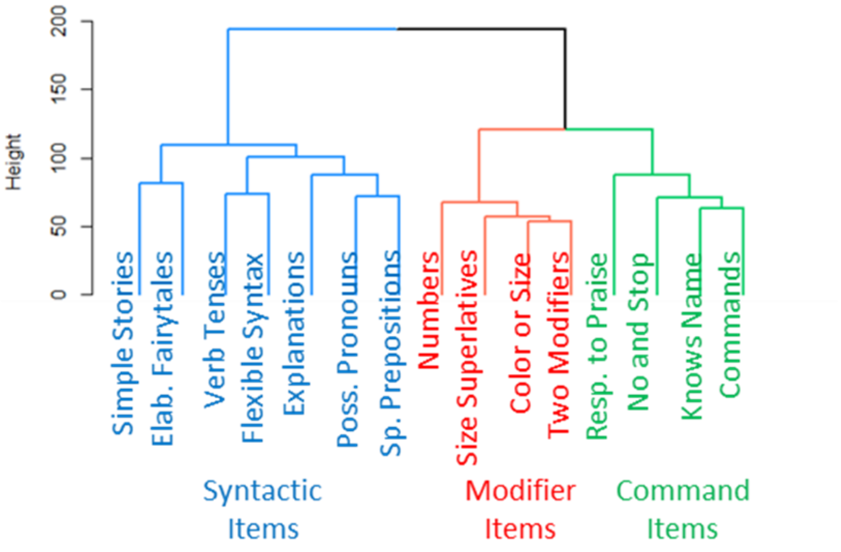

b **Principal Component Analysis**

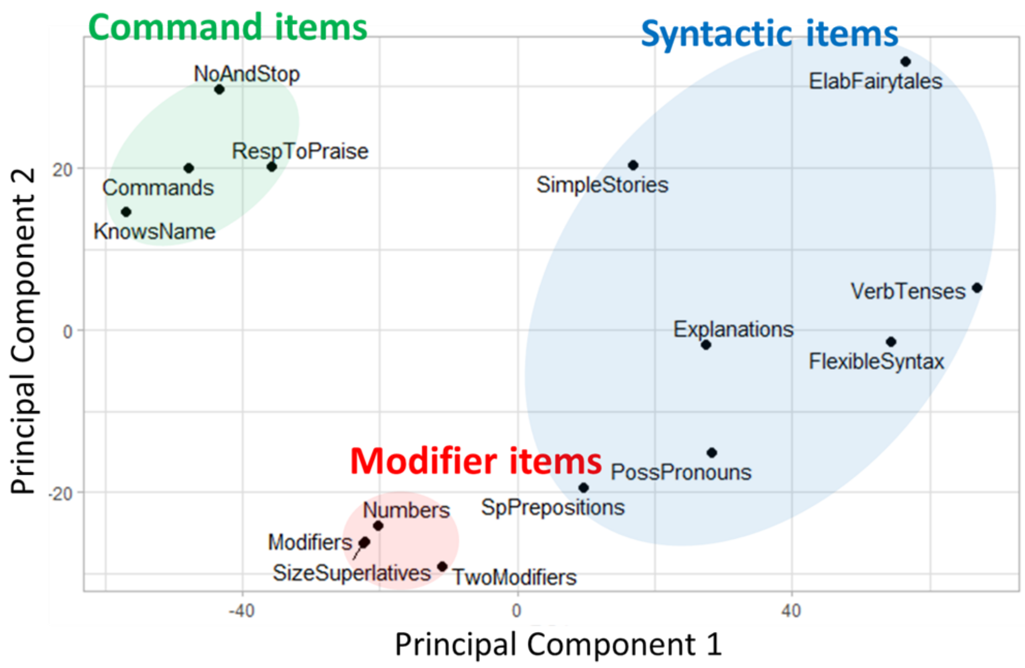

Supplementary Figure 6. Clustering analysis of language comprehension items (**males only**, 12,898 participants, 4 to 22 years of age). (a) The dendrogram representing the hierarchical clustering of language comprehension abilities. (b) Principal component analysis of the 15 language comprehension abilities shows a clear separation between command, modifier, and syntactic items. Principal component 1 accounts for 36.2% of the variance in the data. Principal component 2 accounts for 10.9% of the variance in the data.

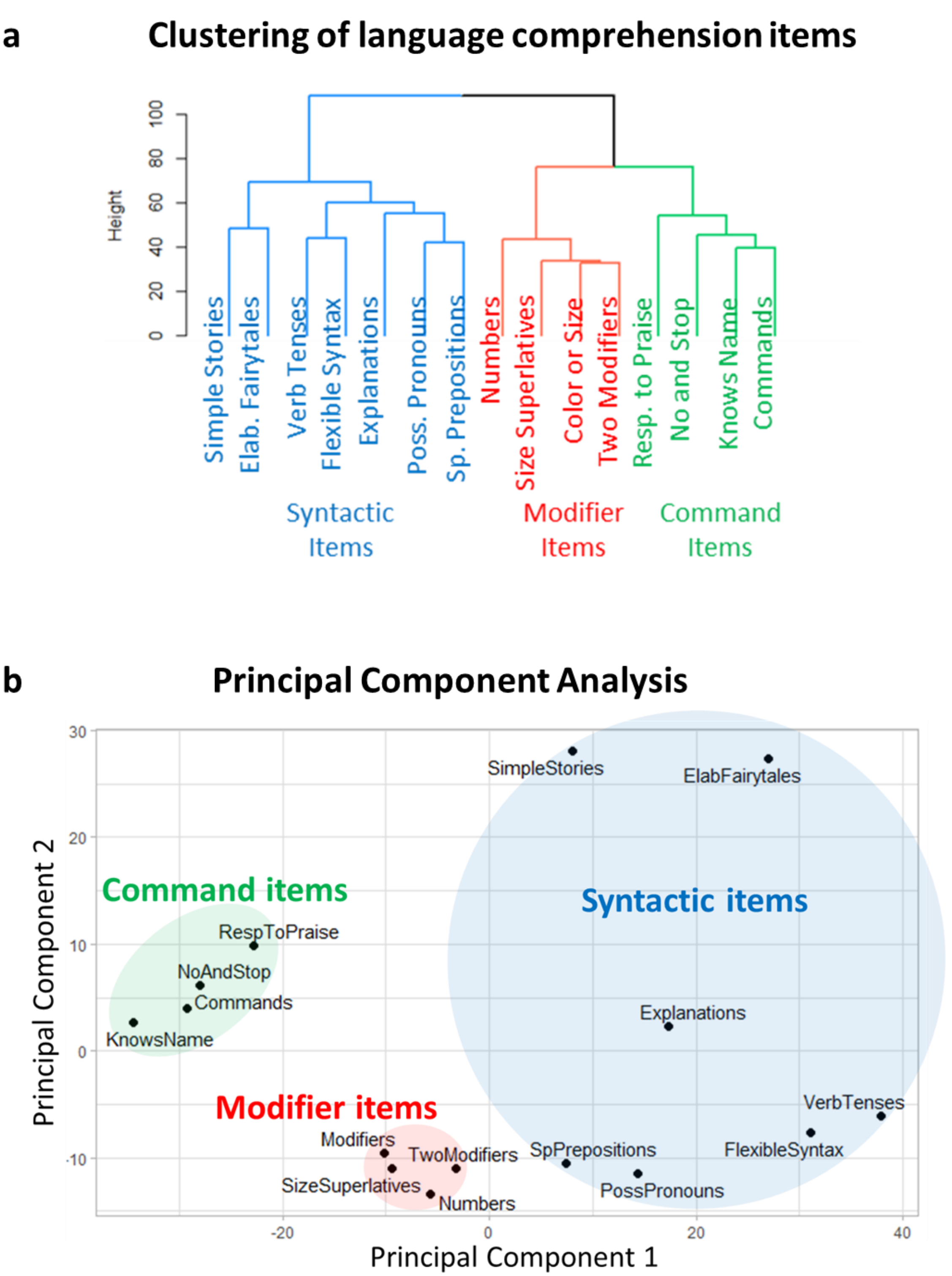

Supplementary Figure 7. Clustering analysis of language comprehension items (**females only**, 4,950 participants, 4 to 22 years of age). (a) The dendrogram representing the hierarchical clustering of language comprehension abilities. (b) Principal component analysis of the 15 language comprehension abilities shows a clear separation between command, modifier, and syntactic items. Principal component 1 accounts for 32.3% of the variance in the data. Principal component 2 accounts for 11.2% of the variance in the data.

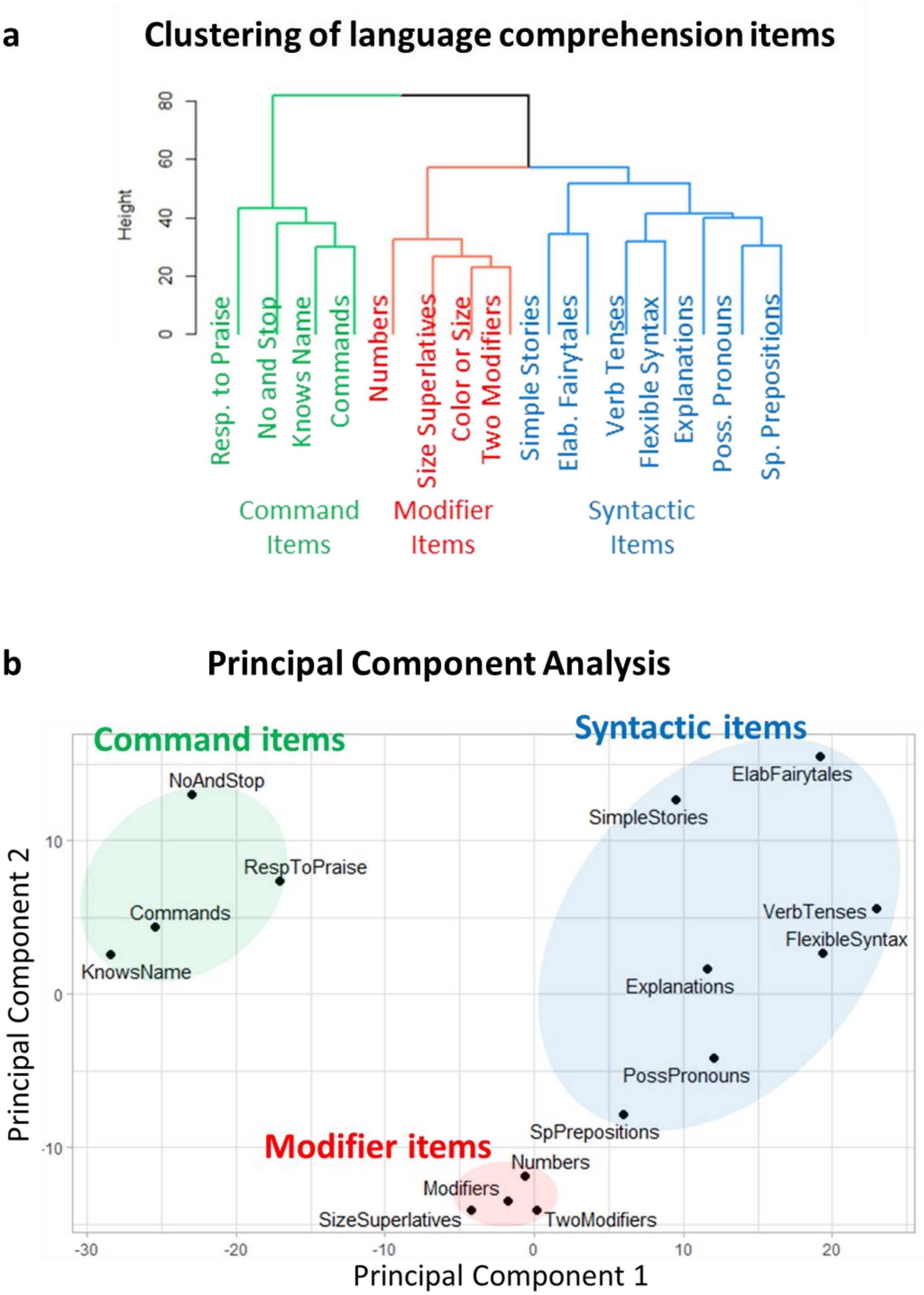

Supplementary Figure 8. Clustering analysis of language comprehension items (**parents with high school education or less**, 2550 participants, 4 to 22 years of age). (a) The dendrogram representing the hierarchical clustering of language comprehension abilities. (b) Principal component analysis of the 15 language comprehension abilities shows a clear separation between command, modifier, and syntactic items. Principal component 1 accounts for 31.1% of the variance in the data. Principal component 2 accounts for 11.7% of the variance in the data.

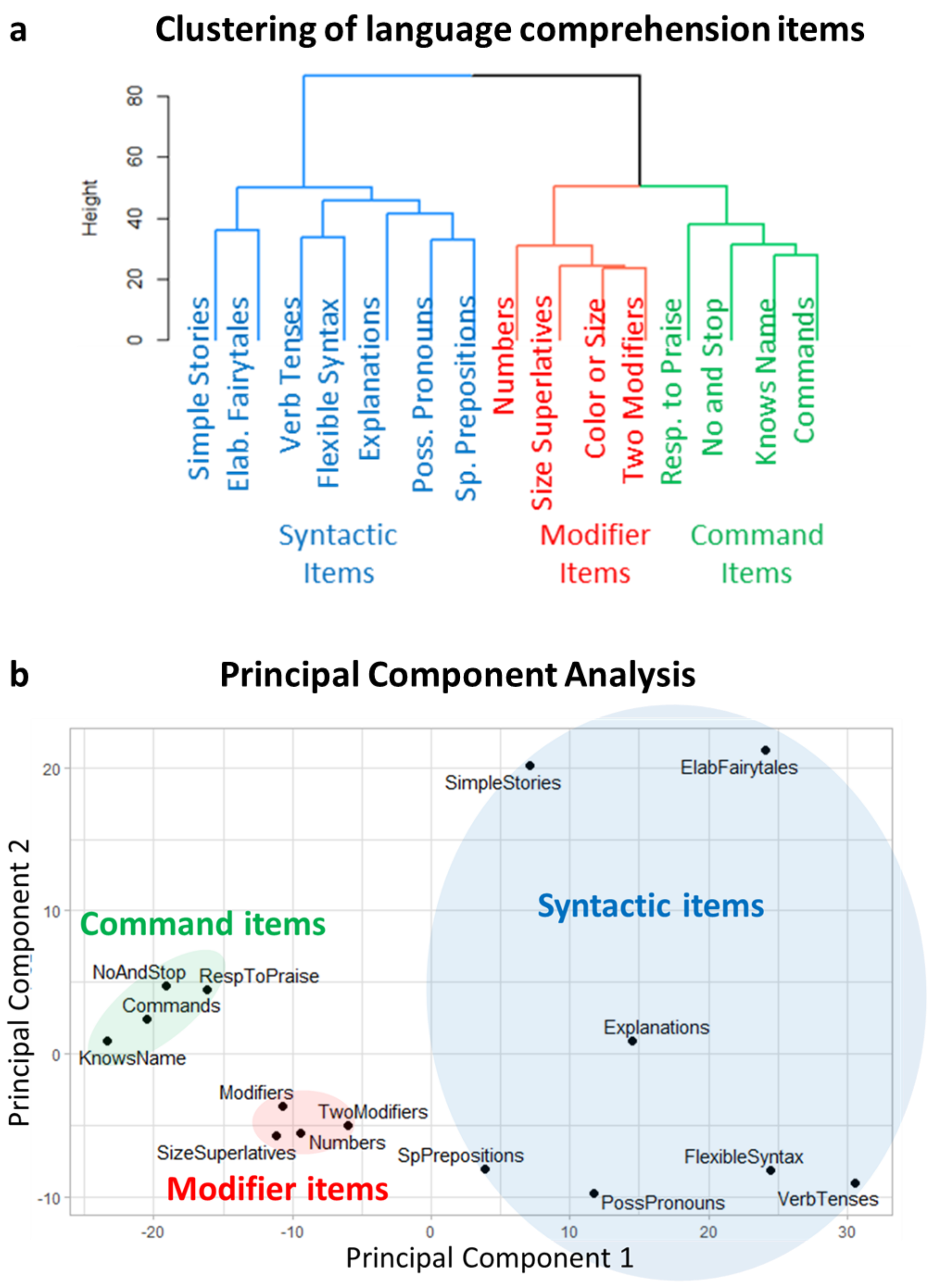

Supplementary Figure 9. Clustering analysis of language comprehension items (**parents with college education**, 2882 participants, 4 to 22 years of age). (a) The dendrogram representing the hierarchical clustering of language comprehension abilities. (b) Principal component analysis of the 15 language comprehension abilities shows a clear separation between command, modifier, and syntactic items. Principal component 1 accounts for 35.6% of the variance in the data. Principal component 2 accounts for 10.5% of the variance in the data.

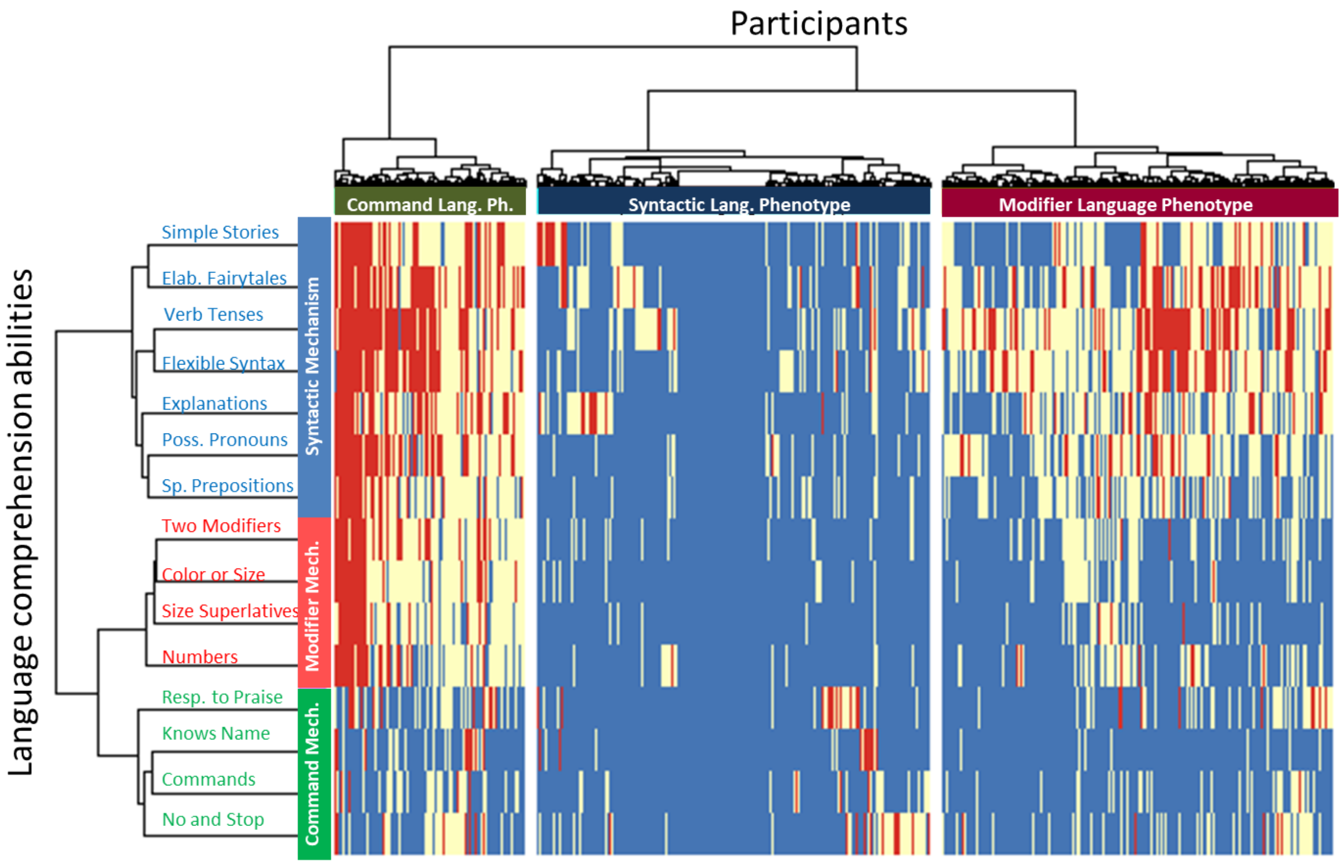

Supplementary Figure 10. Two-dimensional heatmap relating 10,454 participants (4 to 6 years of age) to their language comprehension abilities. The 15 language comprehension abilities are shown as rows. The dendrogram representing language comprehension abilities is shown on the left. Participants are shown as 10,454 columns. The dendrogram representing participants is shown on the top. The green bar labels the command mechanism, the red bar labels the modifier mechanism, and the blue bar labels the syntactic mechanism. The center grid indicates the presence or absence of each ability in each participant: blue signifies the presence of a linguistic ability ("very true" answer), red indicates its absence ("not true" answer), and white represents a partial presence ("somewhat true" answer).

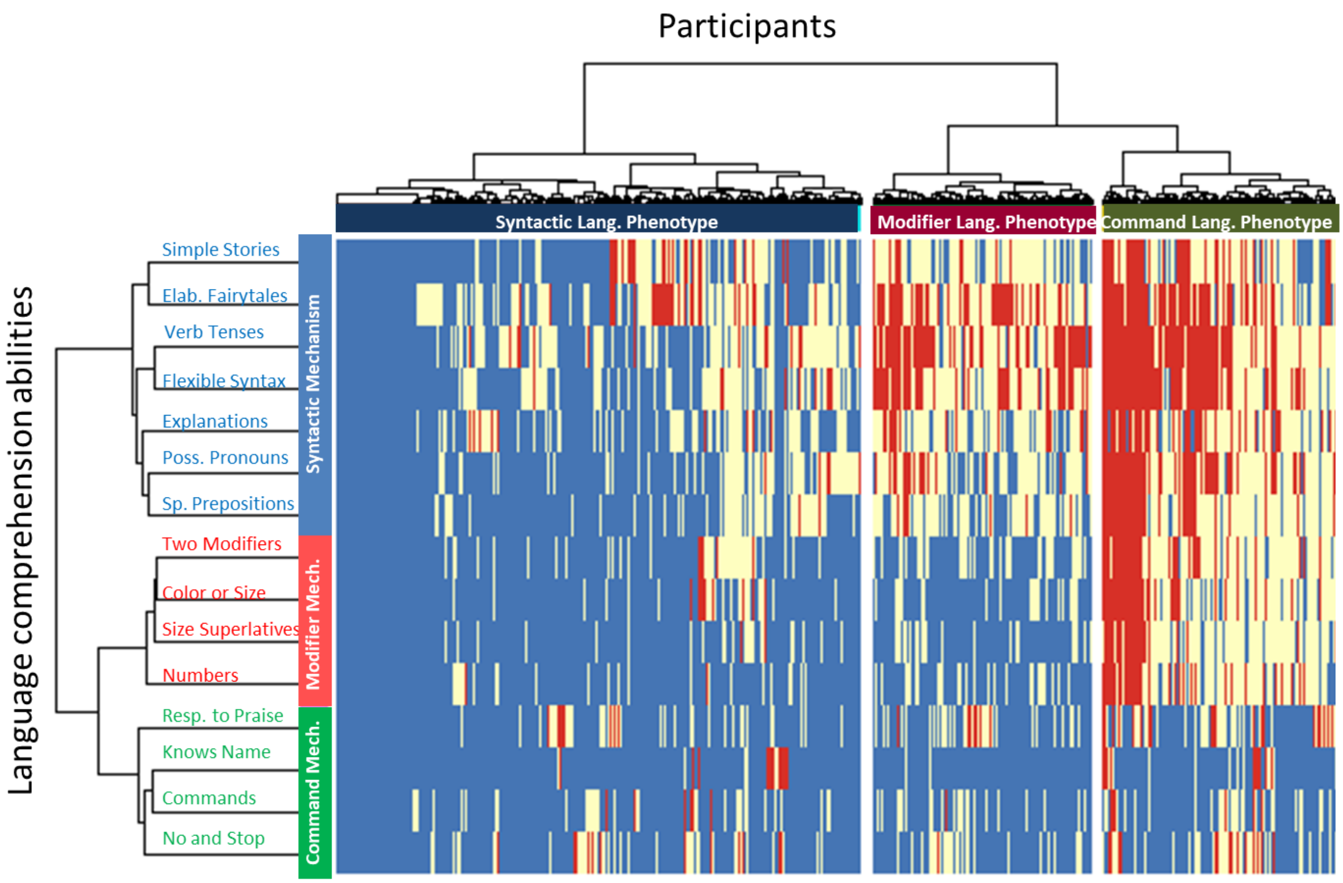

Supplementary Figure 11. Two-dimensional heatmap relating 6,745 participants (6 to 12 years of age) to their language comprehension abilities. The 15 language comprehension abilities are shown as rows. The dendrogram representing language comprehension abilities is shown on the left. Participants are shown as 6,745 columns. The dendrogram representing participants is shown on the top. The green bar labels the command mechanism, the red bar labels the modifier mechanism, and the blue bar labels the syntactic mechanism. The center grid indicates the presence or absence of each ability in each participant: blue signifies the presence of a linguistic ability ("very true" answer), red indicates its absence ("not true" answer), and white represents a partial presence ("somewhat true" answer).

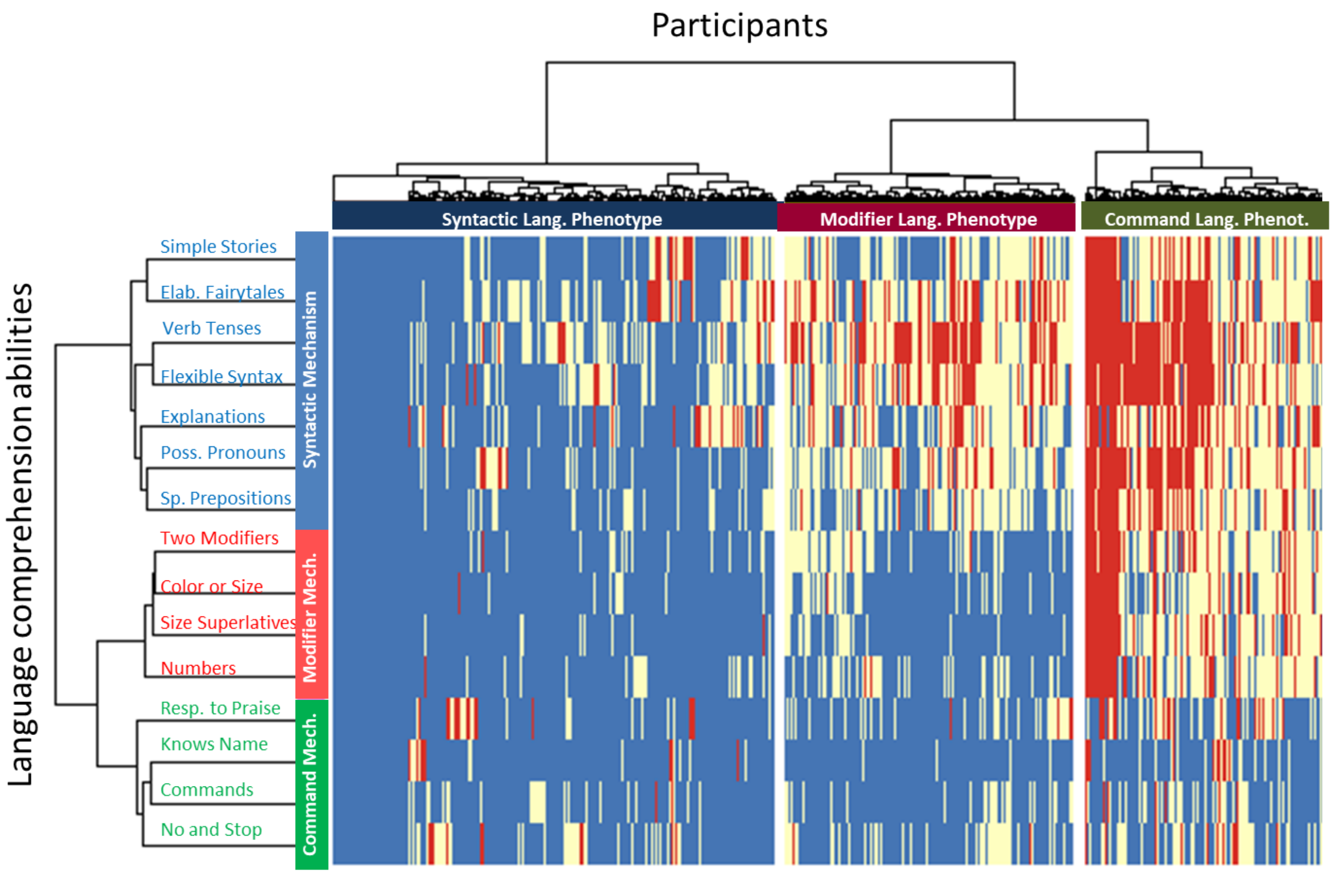

Supplementary Figure 12. Two-dimensional heatmap relating 7,378 participants (6 to 22 years of age) to their language comprehension abilities. The 15 language comprehension abilities are shown as rows. The dendrogram representing language comprehension abilities is shown on the left. Participants are shown as 7,378 columns. The dendrogram representing participants is shown on the top. The green bar labels the command mechanism, the red bar labels the modifier mechanism, and the blue bar labels the syntactic mechanism. The center grid indicates the presence or absence of each ability in each participant: blue signifies the presence of a linguistic ability ("very true" answer), red indicates its absence ("not true" answer), and white represents a partial presence ("somewhat true" answer).

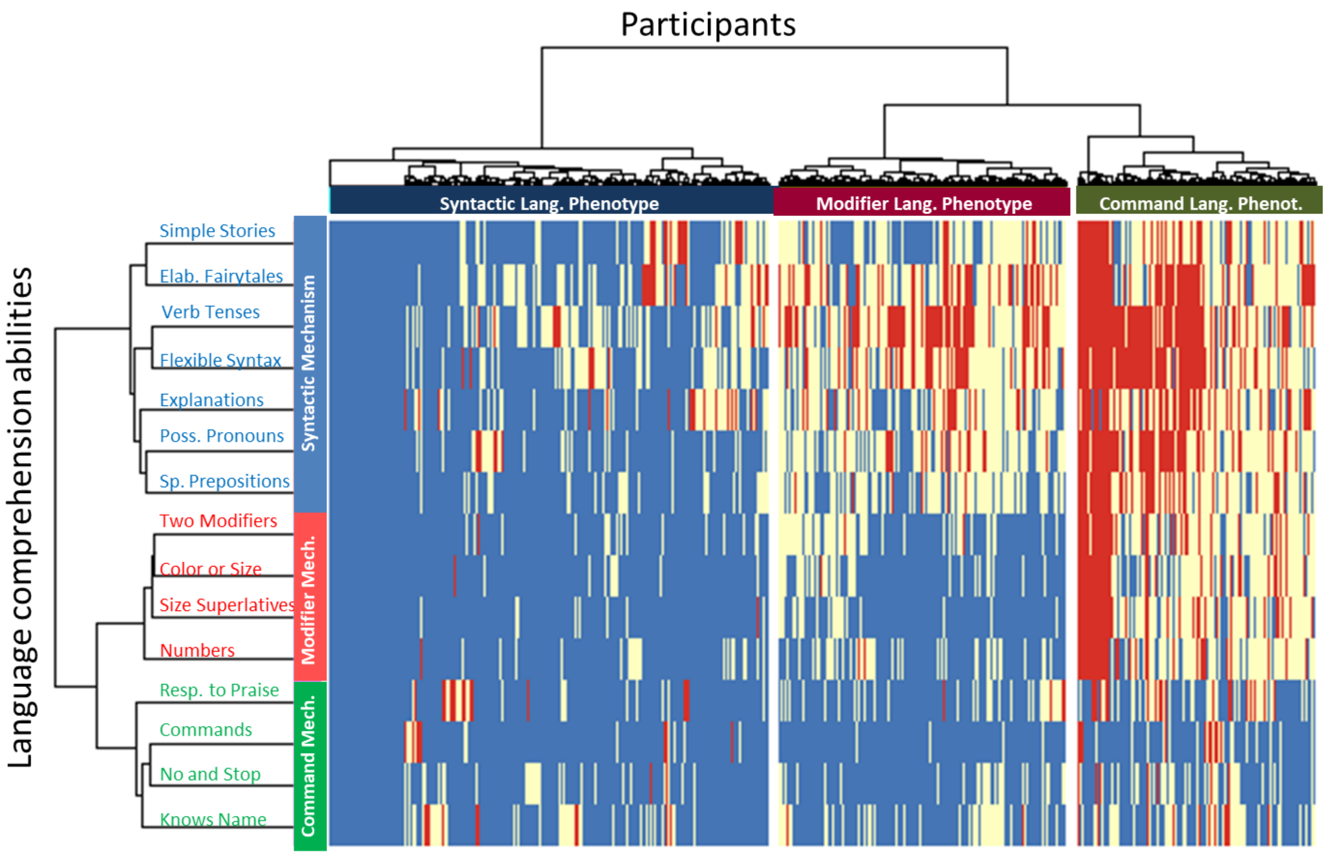

Supplementary Figure 13. Two-dimensional heatmap relating 17,484 participants (4 to 22 years of age, **first evaluation**) to their language comprehension abilities. The 15 language comprehension abilities are shown as rows. The dendrogram representing language comprehension abilities is shown on the left. Participants are shown as 55,558 columns. The dendrogram representing participants is shown on the top. The green bar labels the command mechanism, the red bar labels the modifier mechanism, and the blue bar labels the syntactic mechanism. The center grid indicates the presence or absence of each ability in each participant: blue signifies the presence of a linguistic ability ("very true" answer), red indicates its absence ("not true" answer), and white represents a partial presence ("somewhat true" answer).

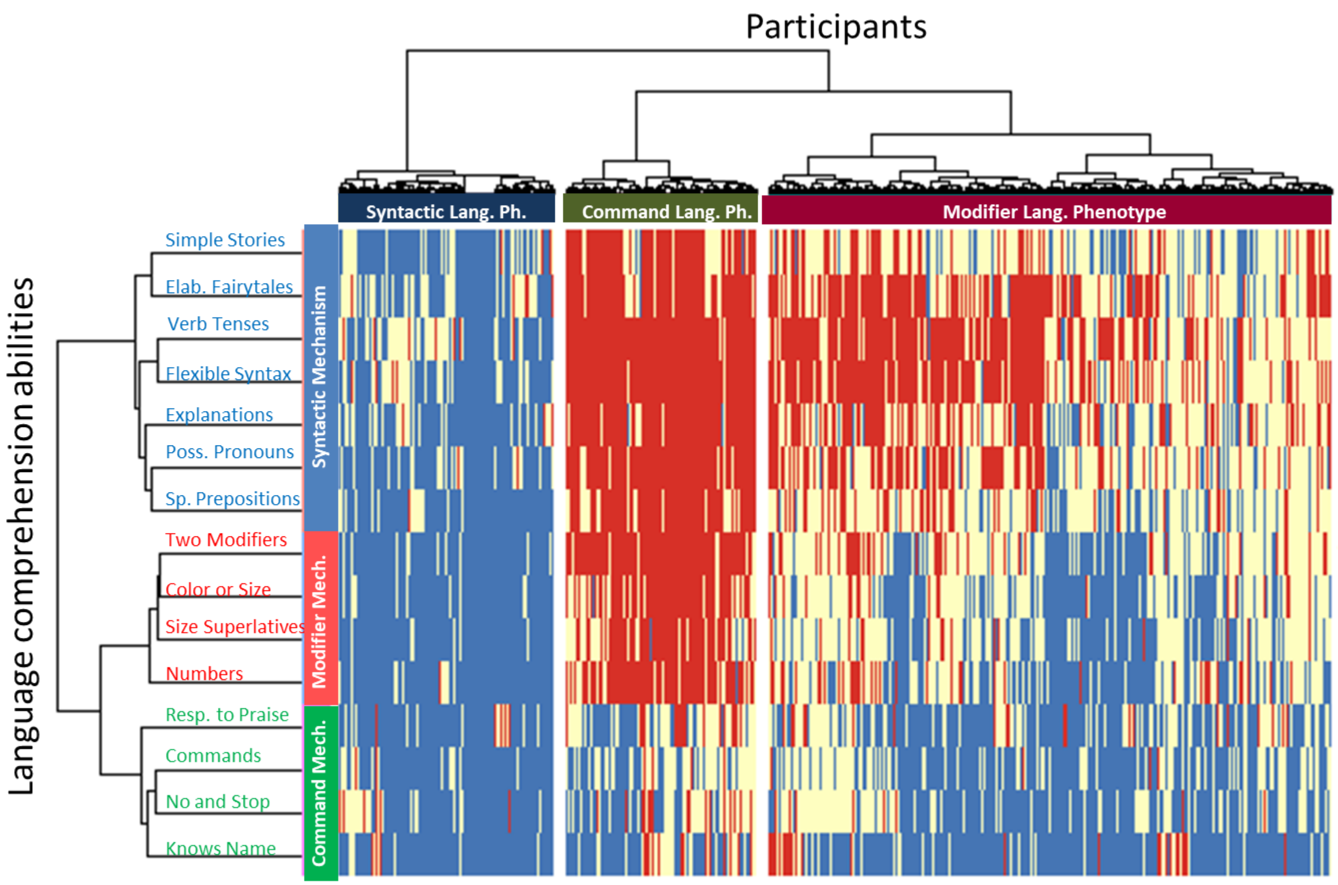

Supplementary Figure 14. Two-dimensional heatmap relating all participants independent of their verbal level (N=55,558) to their language comprehension abilities. The 15 language comprehension abilities are shown as rows. The dendrogram representing language comprehension abilities is shown on the left. Participants are shown as 55,558 columns. The dendrogram representing participants is shown on the top. The green bar labels the command mechanism, the red bar labels the modifier mechanism, and the blue bar labels the syntactic mechanism. The center grid indicates the presence or absence of each ability in each participant: blue signifies the presence of a linguistic ability ("very true" answer), red indicates its absence ("not true" answer), and white represents a partial presence ("somewhat true" answer).

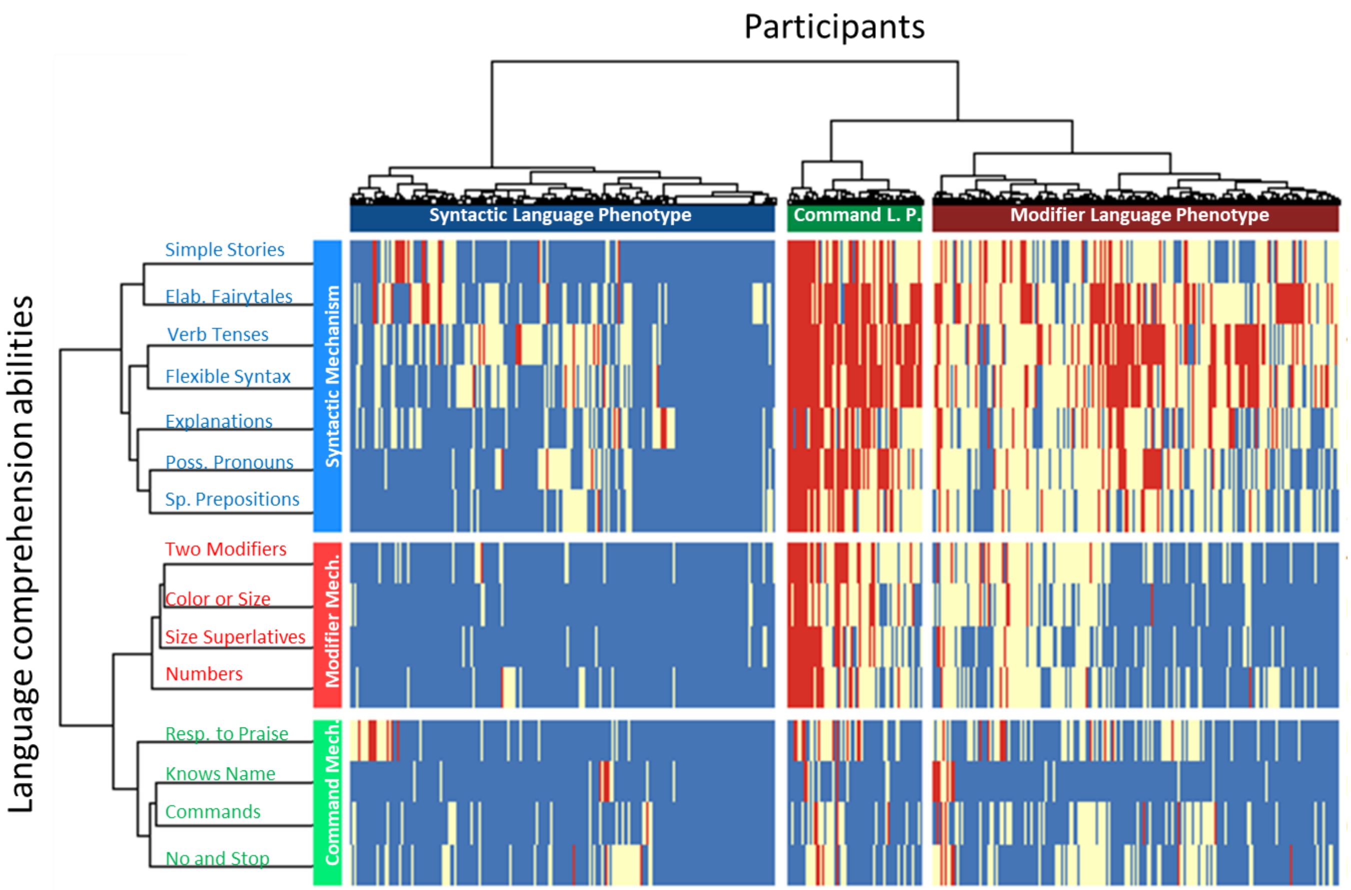

Supplementary Figure 15. Two-dimensional heatmap relating **male participants** (N=12,898, 4 to 22 years of age) to their language comprehension abilities. The 15 language comprehension abilities are shown as rows. The dendrogram representing language comprehension abilities is shown on the left. Participants are shown as 12,898 columns. The dendrogram representing participants is shown on the top. The green bar labels the command mechanism, the red bar labels the modifier mechanism, and the blue bar labels the syntactic mechanism. The center grid indicates the presence or absence of each ability in each participant: blue signifies the presence of a linguistic ability (“very true” answer), red indicates its absence (“not true” answer), and white represents a partial presence (“somewhat true” answer).

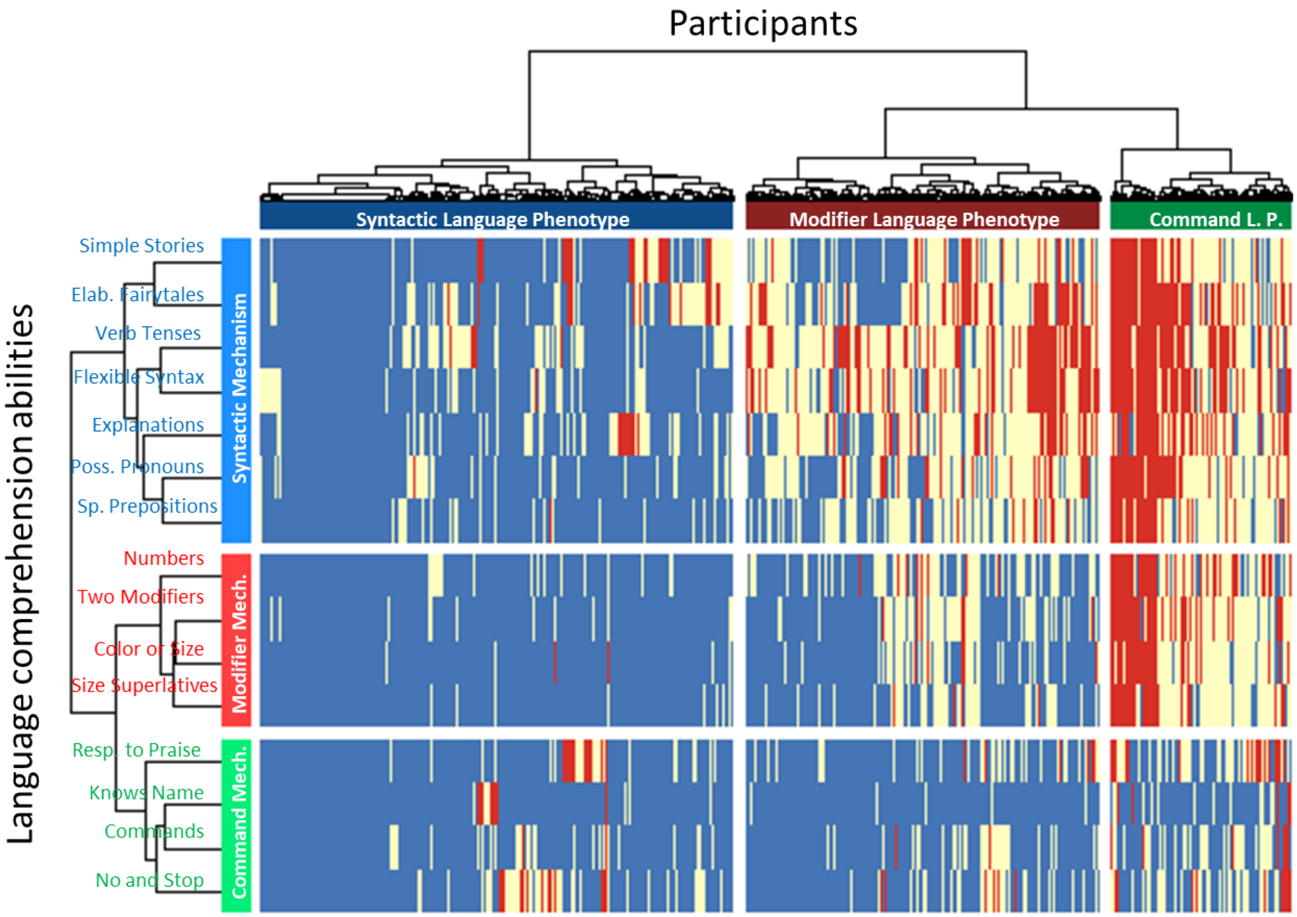

Supplementary Figure 16. Two-dimensional heatmap relating **female participants** (N=4,950, 4 to 22 years of age) to their language comprehension abilities. The 15 language comprehension abilities are shown as rows. The dendrogram representing language comprehension abilities is shown on the left. Participants are shown as 4,950 columns. The dendrogram representing participants is shown on the top. The green bar labels the command mechanism, the red bar labels the modifier mechanism, and the blue bar labels the syntactic mechanism. The center grid indicates the presence or absence of each ability in each participant: blue signifies the presence of a linguistic ability ("very true" answer), red indicates its absence ("not true" answer), and white represents a partial presence ("somewhat true" answer).

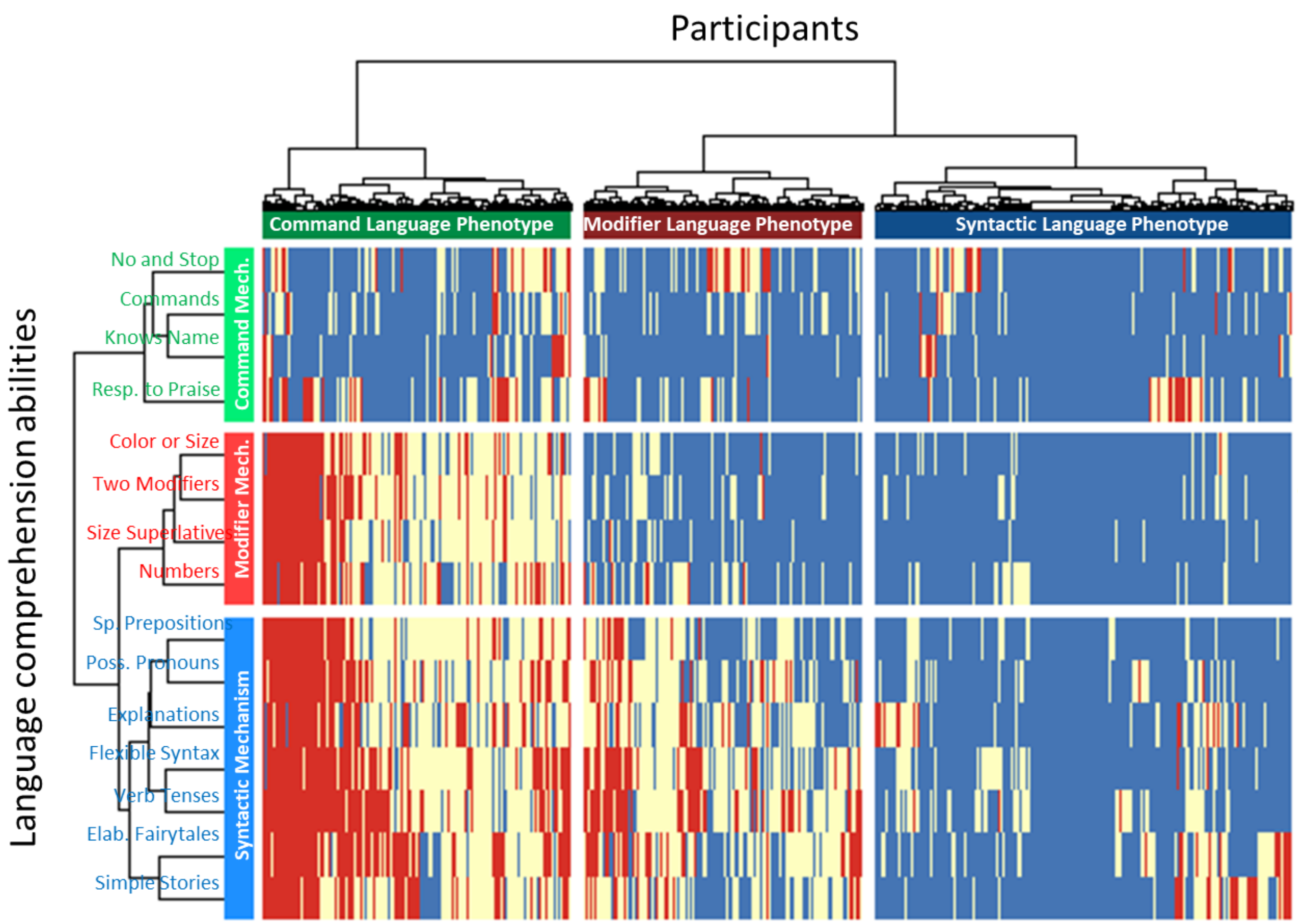

Supplementary Figure 17. Two-dimensional heatmap relating participants whose **parental education was high school or less** (N= 2550, 4 to 22 years of age) to their language comprehension abilities. The 15 language comprehension abilities are shown as rows. The dendrogram representing language comprehension abilities is shown on the left. Participants are shown as 2,550 columns. The dendrogram representing participants is shown on the top. The green bar labels the command mechanism, the red bar labels the modifier mechanism, and the blue bar labels the syntactic mechanism. The center grid indicates the presence or absence of each ability in each participant: blue signifies the presence of a linguistic ability ("very true" answer), red indicates its absence ("not true" answer), and white represents a partial presence ("somewhat true" answer).

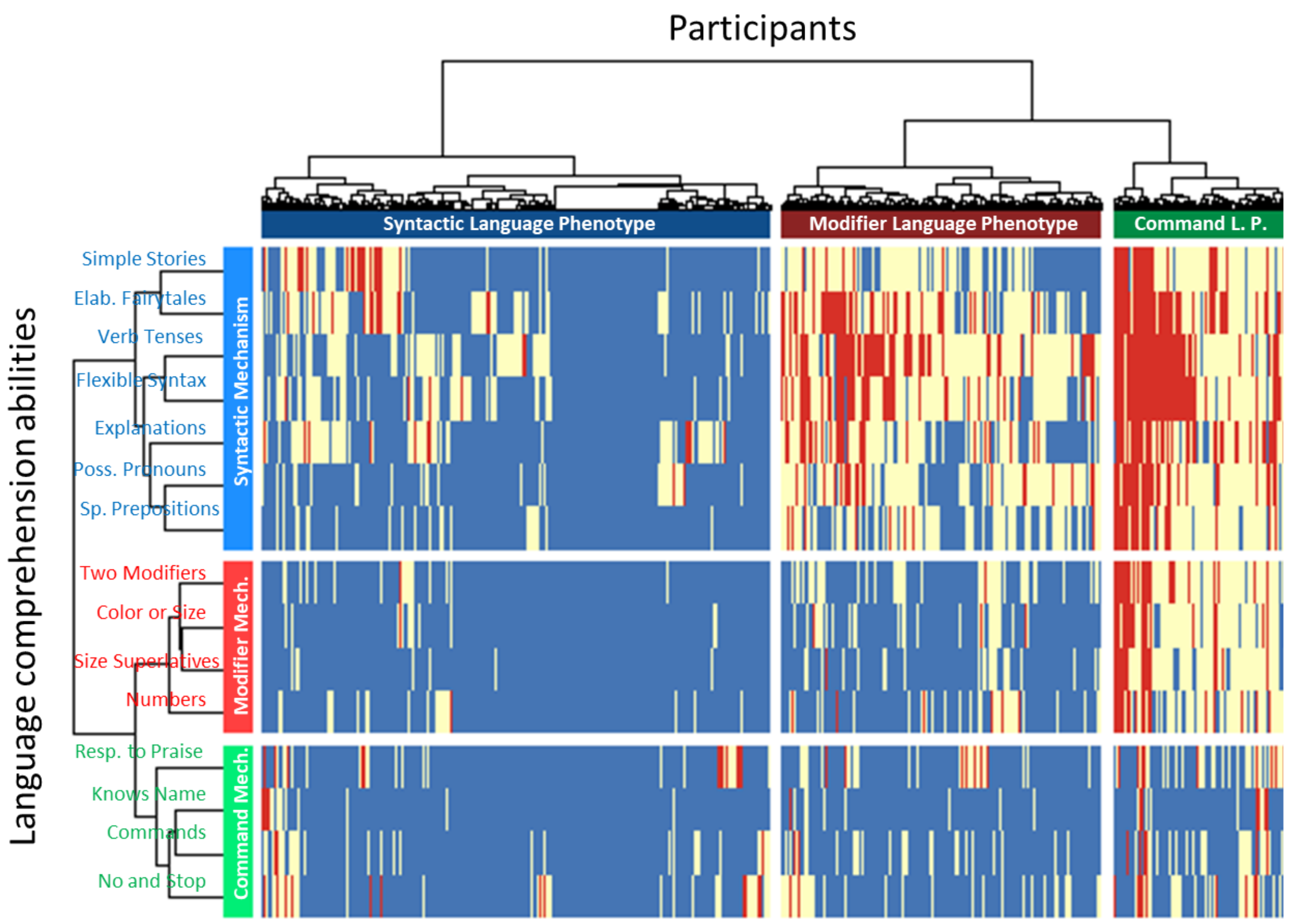

Supplementary Figure 18. Two-dimensional heatmap relating participants whose **parents graduated college** (N= 2882, 4 to 22 years of age) to their language comprehension abilities. The 15 language comprehension abilities are shown as rows. The dendrogram representing language comprehension abilities is shown on the left. Participants are shown as 2,882 columns. The dendrogram representing participants is shown on the top. The green bar labels the command mechanism, the red bar labels the modifier mechanism, and the blue bar labels the syntactic mechanism. The center grid indicates the presence or absence of each ability in each participant: blue signifies the presence of a linguistic ability ("very true" answer), red indicates its absence ("not true" answer), and white represents a partial presence ("somewhat true" answer).

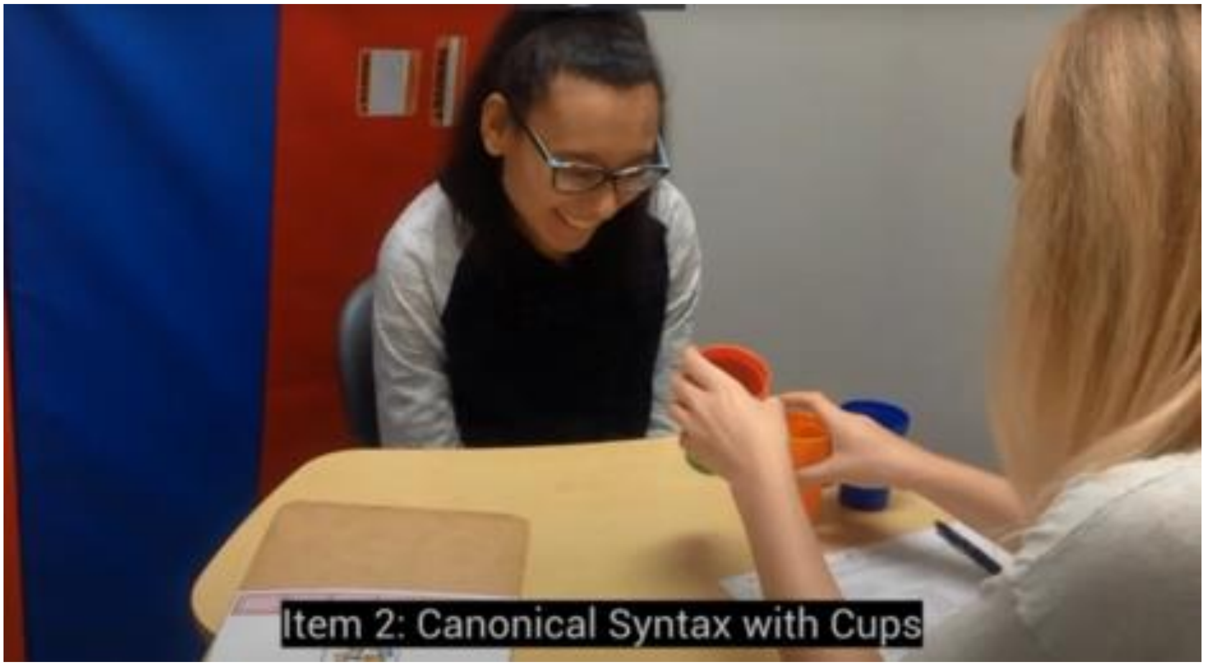

Supplementary Movie 1. Reprinted with permission from [3]. Authors have obtained written parental consent to publish the video. Most ASD participants were able to complete the canonical stacking cups task (e.g., "put the red cup inside the green cup"), but were unable to complete the same task under the condition of non-canonical word order ("inside the green cup, put the red cup"). Failing ASD participants usually selected the correct cups, but assembled them randomly. https://youtu.be/Hh7pkZB4ETU

a Hierarchical clustering of language comprehension items

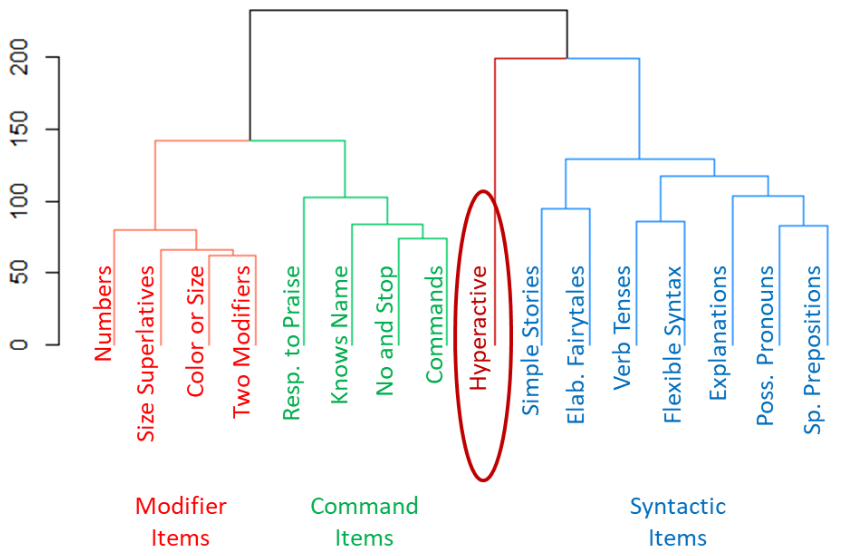

b Principal Component Analysis

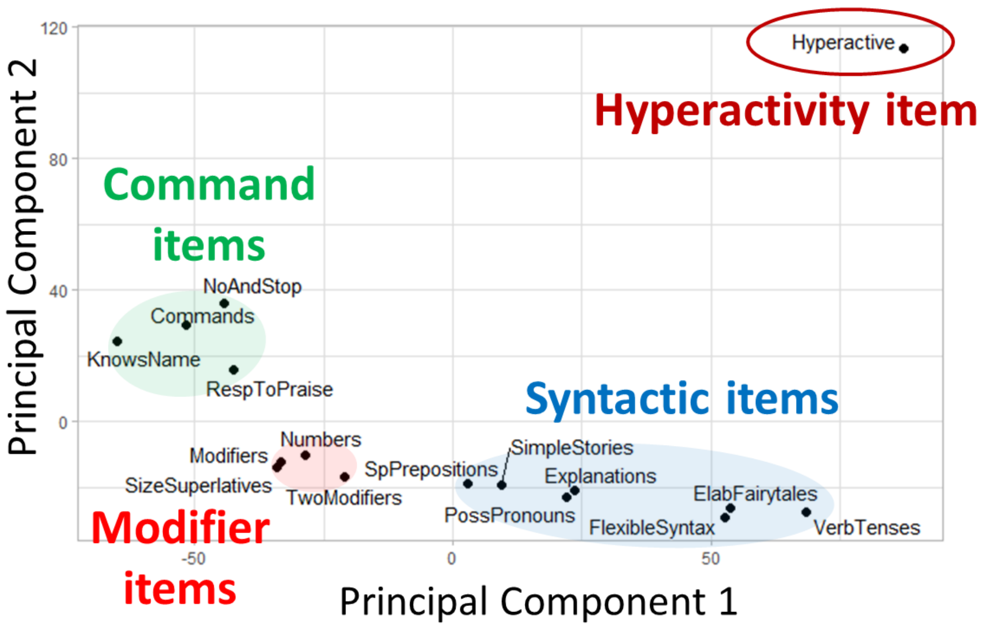

Supplementary Figure 19. Clustering analysis of language comprehension items (17,848 participants, 4 to 22 years of age, first evaluation). (a) The dendrogram representing the hierarchical clustering of language comprehension abilities. (b) Principal component analysis of the 15 language comprehension abilities shows a clear separation between command, modifier, and syntactic items. Principal component 1 accounts for 34.3% of the variance in the data. Principal component 2 accounts for 10.9% of the variance in the data.

The language therapy app used to collect data

The app includes language exercises aiming to develop syntactic language. The app verbal activities use higher forms of language, such as noun-adjective combinations, spatial prepositions, recursion, and syntax: e.g., a child can be instructed to put the *large red dog behind the orange chair*, Supplementary Figure 20; or *take animals home* following an explanation that *the lion lives above the monkey and under the cow*, Supplementary Figure 21. In every activity a child listens to a short story and then works within an immersive interface to generate an answer. Correct answers are rewarded with pre-recorded encouragement and flying stars. To avoid routinization, all instruction sentences are generated dynamically, assembled from individual pre-recorded words. For example, in Figure 21, the instruction rotates through many different animals: *the* {*lion | bunny | lion | dog | cat*} *lives above the* {*monkey | horse | bear | elephant | giraffe*} *and under the* {*cow | duck | dinosaur | turtle | sheep | crocodile*} and many different variations of sentence structure. Collectively, activities have over 10 million different instructions, therefore a child will almost never hear the same instruction twice.

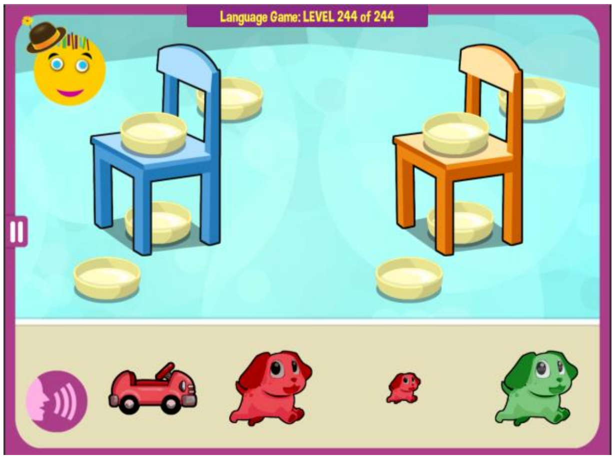

Supplementary Figure 20. Examples of exercises: a child is instructed: *Put the large red dog behind the orange chair.*

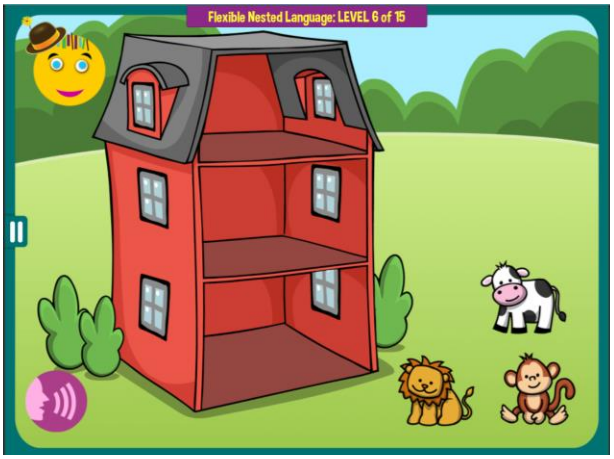

Supplementary Figure 21. a child is instructed: Imagine. *The lion lives above the monkey and under the cow. Take animals home.*

Note that pictures cannot be dragged to their targets during instructions, encouraging a child to listen, remember, process, and plan their movement mentally.

The app's nonverbal activities aim to train syntactic language visually through implicit instructions. For example, a child can be presented with two separate images of a train and a window pattern, and a choice of complete trains. The task is to find the correct complete train. The child is encouraged to avoid trial-and-error and integrate separate train parts mentally, Supplementary Figure 22. Different games use various tasks and visual patterns to keep the child engaged, Supplementary Figure 23. Most puzzles are assembled dynamically from multiple pieces in such a way that they never repeat themselves.

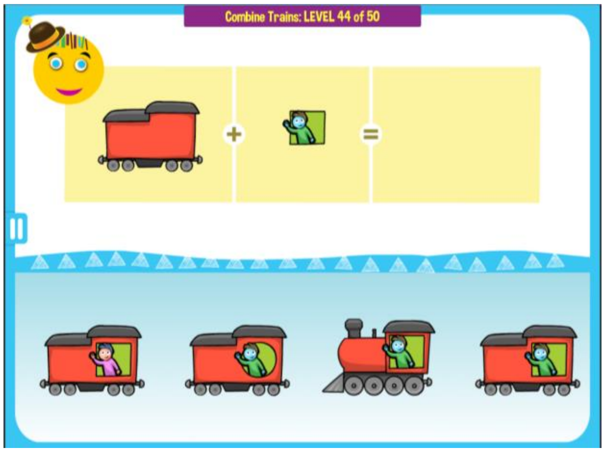

Supplementary Figure 22. Integrate separate train parts mentally.

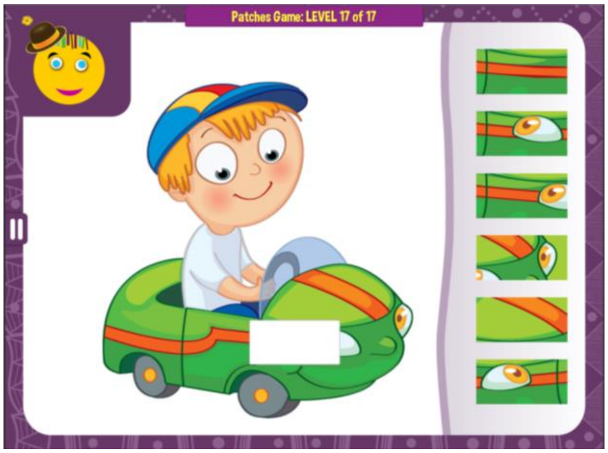

Supplementary Figure 23: Find the correct patch.

The app also includes a number of hybrid activities that start children on easier nonverbal exercises and then gradually increase in difficulty, first to a combination of a verbal instruction and a visual clue and later to a verbal instruction alone, Supplementary Figures 24, 25.

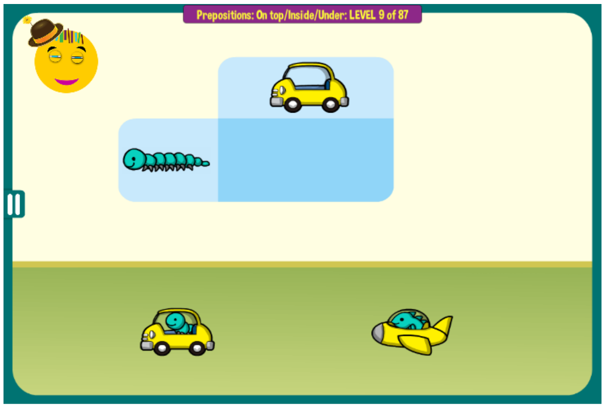

Supplementary Figure 24. Examples of a game that teach spatial prepositions *above* and *under.* The game starts with the implicit instruction to combine an animal and a vehicle.

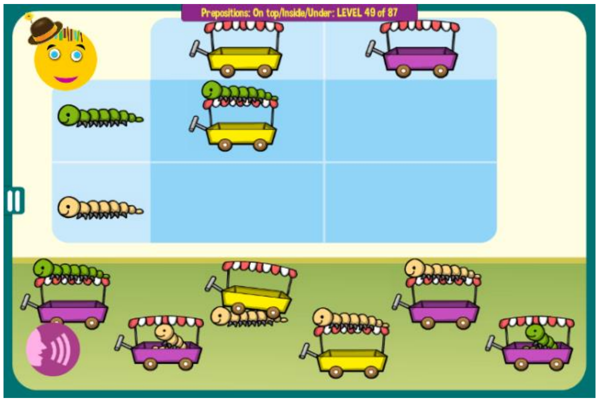

Supplementary Figure 25. At more difficult levels a child must notice the correct positioning of the animal (above or under), which is announced verbally and also indicated by a visual clue in the top left corner of the matrix (in this figure the visual cue is the caterpillar on top of the cart). At the most difficult levels (not shown) the visual clue is hidden and a child must rely on the verbal instruction alone.

Supplementary References

1. Rimland, B. & Edelson, S. M. Autism treatment evaluation checklist (ATEC). *Autism Res. Inst. San Diego CA* (1999).
2. Braverman, J., Dunn, R. & Vyshedskiy, A. Development of the Mental Synthesis Evaluation Checklist (MSEC): A Parent-Report Tool for Mental Synthesis Ability Assessment in Children with Language Delay. *Children* **5**, 62 (2018).
3. Vyshedskiy, A. Imagination in Autism: A Chance to Improve Early Language Therapy. *Healthcare* **9**, 63 (2021).
